# The GPI Anchor Signal Sequence Dictates the Folding and Functionality of the Als5 Adhesin from *Candida albicans*

**DOI:** 10.1371/journal.pone.0035305

**Published:** 2012-04-11

**Authors:** Mohammad Faiz Ahmad, Bhawna Yadav, Pravin Kumar, Amrita Puri, Mohit Mazumder, Anwar Ali, Samudrala Gourinath, Rohini Muthuswami, Sneha Sudha Komath

**Affiliations:** 1 School of Life Sciences, Jawaharlal Nehru University, New Delhi, India; 2 Department of Chemistry, Jamia Millia Islamia, New Delhi, India; 3 Department of Plant Molecular Biology, University of Delhi, New Delhi, India; 4 Invisible Sentinel, Biotech Company, Philadelphia, Pennsylvania, United States of America; David Geffen School of Medicine at University of California Los Angeles, United States of America

## Abstract

**Background:**

Proteins destined to be Glycosylphosphatidylinositol (GPI) anchored are translocated into the ER lumen completely before the C-terminal GPI anchor attachment signal sequence (SS) is removed by the GPI-transamidase and replaced by a pre-formed GPI anchor precursor. Does the SS have a role in dictating the conformation and function of the protein as well?

**Methodology/Principal Findings:**

We generated two variants of the Als5 protein without and with the SS in order to address the above question. Using a combination of biochemical and biophysical techniques, we show that in the case of Als5, an adhesin of *C. albicans*, the C-terminal deletion of 20 amino acids (SS) results in a significant alteration in conformation and function of the mature protein.

**Conclusions/Significance:**

We propose that the locking of the conformation of the precursor protein in an alternate conformation from that of the mature protein is one probable strategy employed by the cell to control the behaviour and function of proteins intended to be GPI anchored during their transit through the ER.

## Introduction

A wide variety of proteins are known to be anchored to the extra-cytoplasmic leaflet of the plasma membrane by glycosylphosphatidylinositol (GPI) anchors and defects in GPI anchor attachment can have severe consequences for the eukaryotic cell [1]. Proteins destined to be GPI anchored possess a C-terminal signal sequence specific for this modification [2]. Unlike integral membrane proteins that have their transmembrane domains co-translationally inserted into the membrane via the translocon pore, proteins meant to be GPI anchored are completely translocated into the ER lumen [3]. Shortly thereafter, these are acted upon by the GPI-transamidase and have their C-terminal GPI anchor attachment signal sequence (SS) replaced by a pre-formed GPI anchor.

Is the role of the SS confined to being a signal for GPI anchor attachment or does it also control the conformation and function of a protein destined to be GPI anchored? In order to address this question, we chose to study Als5, an adhesin from *Candida albicans*.

*ALS5* belongs to the agglutinin-like sequence (*ALS)* family of genes which code for eight adhesins in *Candida albicans*. These adhesins are important for establishment of commensal colonies of the organism in the host as well as in its pathogenesis and virulence under appropriate conditions [4]. Since they are tethered to the membrane via GPI anchors, any defects in GPI anchor biosynthesis can drastically affect the pathogenesis and virulence of the organism [5]–[7]. Indeed, complete GPI anchors have been shown to be important for morphogenesis, virulence and macrophage-resistance of the organism [7].

Like other members of the Als family of adhesins, Als5 has an N-terminal secretion signal followed by a large immunoglobulin-like domain, a highly conserved Thr-rich segment, a central domain containing variable numbers of tandem repeats of Ser/Thr sequences, a C-terminal Ser/Thr rich stalk and the C-terminal signal sequence for GPI anchor attachment [8]. When heterologously expressed in *S. cerevisiae*, Als5 can make the host cells adhere to basal lamina proteins such as collagen type IV and fibronectin [9]. The protein has also been shown to be capable of mediating endothelial cell invasion and its N-terminal domain has been shown to be important for adherence [10], [11]. The protein has a tendency to aggregate and form amyloid-like fibrils; a potential amyloidogenic domain has also been identified [11]–[13] .

In this study, we show that it is possible to express Als5 as a GST-fusion protein in bacterial cells and to purify it using affinity chromatography. We show that the Als5 protein thus purified is capable of adhering to collagen and forming self-aggregates, and is therefore ‘functional’. In contrast, we show that the Als5-SS variant, possessing the GPI achor attachment signal sequence, poorly binds to collagen type IV and does not form aggregates. We attribute this to the differences in the secondary structure of the two proteins. The implications of these results are discussed in the context of the cell.

## Materials and Methods

### Materials

All chemicals were of analytical grade and were purchased either from Qualigens, Merck, SRL or Sigma-Aldrich (USA). Components of media were purchased from Himedia (India); DH5α and BL21(DE3) cells as well as glutathione-agarose beads from Bangalore Genei; PreScission^TM^ protease and pGEX-6P-2 from GE-Healthcare; restriction enzymes as well as DNA and protein molecular weight markers from MBI Fermentas; collagen type IV from Sigma; gel extraction kit from Qualigens; anti-GST antibodies were from Santa Cruz, protease cocktail inhibitor (P8340) from Sigma; N-gycosidase F from Roche. Peptide synthesis was carried out by Custom Peptide Synthesis service of USV Ltd. (India). The primers (Table S1) were custom-synthesized by Sigma-Aldrich.

### Cloning of *ALS5* Gene with and without the C-terminal GPI Anchor Attachment Signal Sequence for Expression in *E.coli*

We began with cloning, expression and purification of Als5 protein from the CAI4 strain (a derivative of SC5314, *ura3Δ::imm434/ura3Δ::imm434*) of *Candida albicans*. This strain has two different alleles for Als5, varying in the number of sequences coding for the tandem repeats. The one corresponding to the smaller allelic variant of *ALS5* was used for this study. Full length *ALS5* gene minus the GPI anchor attachment signal sequence (3985–4044 bp), was amplified from the *C. albicans* CAI4 strain using PCR (Table S1), with Pfu polymerase and ligated into the pGEX-6P-2 vector, containing the GST affinity tag, between the BamHI and XhoI sites. The ligation product was transformed into competent DH5α cells and colonies were screened by colony PCR. Plasmid was extracted from the PCR-positive colonies, and the clone confirmed by restriction digestion of the plasmid by BamHI and XhoI enzymes.

DNA sequence analysis confirmed the sequence of the cloned *ALS5* gene as compared to the reported *ALS5* sequence in the Candida genome database (www.candidagenome.org). The protein expressed from this construct is referred to here as GST-Als5.

Similarly, *ALS5-*SS, an *ALS5* variant, with the GPI-anchor attachment signal sequence was cloned into the pGEX-6P-2 vector under the BamHI and XhoI sites. The protein expressed from this construct is referred to as GST-Als5-SS.

### Expression and Purification of GST-Als5 and GST-Als5-SS Proteins

The expression of the GST-Als5 and the GST-Als5-SS proteins were optimized with respect to IPTG concentration, induction period and temperature. Thus, we induced protein expression at 16^o^C for 6 hours using 0.1 mM IPTG concentration.

The cell pellets were resuspended in lysis buffer (10 µM PMSF, 150 mM NaCl, 50 mM sodium phosphate buffer (pH 8.0), 5% glycerol, 0.1 mg/ml lysozyme, 1∶100 diluted protease inhibitor cocktail), incubated at 4°C for 1 hour, then sonicated (7 cycles, 30s ON, 30s OFF). The cell lysate obtained was centrifuged at 8500 rpm for 1 hour to recover the supernatant, which was loaded onto pre-equilibrated glutathione-agarose beads and incubated for 3 hours at 4°C on a rocker. This was followed by extensive washing of the beads with wash buffer [50 mM sodium phosphate buffer (pH 8.0), 3 M NaCl]. The protein was then eluted with elution buffer (50 mM sodium phosphate buffer (pH 8.0), 150 mM NaCl, 20% glycerol, 10 mM glutathione).

### Western Blots

The purified fractions of GST-Als5 and GST-Als5-SS were confirmed by Western blotting, using polyclonal anti-GST antibody or anti-Als5 antibody as the primary antibody for detection of the GST-tagged proteins. The binding of primary antibody was detected by the HRP-conjugated secondary antibody. The presence of secondary antibody on the blot was detected by using diaminobenzidine as a substrate for HRP.

### Anti-Als5antibodies

The production of the antibodies was outsourced (Merck India Ltd.). Polyclonal antibodies were generated in rabbit against the protein band obtained after an SDS-PAGE run of GST- Als5-SS. The antibodies detected both GST-Als5 as well as GST-Als5-SS on a Western Blot. The specificity of the generated anti-Als5 antibody was checked by detecting its ability to bind to the Candida cell surface adhesins. For staining, Candida SC5314 cells (a kind gift from Prof. Rajendra Prasad, SLS, JNU) were grown in synthetic dextrose minimal medium at 30°C to an OD_600nm_ of 0.5. 500 µl of the cells were taken in an eppendorf and washed with PBS. The cells were then incubated with anti-Als5 primary antibody (diluted1∶1,000 in 1% skimmed milk in PBS, 0.05% Tween-20) for 1 hour at room temperature. The cells were then washed thrice with PBS and incubated for 1 hour with TRITC labelled anti-rabbit secondary antibody (diluted 1∶5000 times in 1% skimmed milk in PBS, 0.05% Tween-20) for 1 hour at room temperature. The cells were then washed thrice with PBS, and the presence of the TRITC labelled secondary Ab on the Candida cells was detected using flow cytometry. To rule out the non-specific interaction of the secondary Ab with the proteins on Candida cell surface, unstained cells were also detected by flow cytometer. In the unstained set, the cells were incubated with the buffer alone used for the primary antibody instead of the anti-Als5 primary Ab, and were processed otherwise similarly to the stained set (mentioned above). Further, the specificity of the primary antibody to bind to Candida cell surface Als5 was checked by the ability of the purified GST-Als5 to inhibit the binding of the anti-Als5 Ab on the Candida cell surface. For this, the cells were incubated with purified GST-Als5 prior to the incubation with anti-Als5 Ab, and were processed otherwise similarly to the stained set. As a control, experiment with GST was also similarly performed. Different concentrations of both the proteins were used to establish the specificity of the anti-Als5 Ab interaction with the Candida cell surface Als5.

### Mass Spectrometric Analysis

The purified GST-Als5 and GST-Als5-SS proteins were analysed for intact mass by MALDI-TOF (Bruker). Further, for confirmation of the identity of the proteins, in-gel tryptic digestion was carried out and the peptide fragments analysed by MALDI-TOF.

### Secondary Structure Studies Using Circular Dichroism (CD) Spectra

The secondary structure analysis of freshly purified GST-Als5 and GST-Als5-SS proteins was done by CD spectroscopy. The concentrations of the proteins were determined using Bradford reagent. The proteins were then dialysed against 50 mM potassium phosphate buffer (pH 8.0), containing 150 mM KCl and 20% glycerol. After dialysis and centrifugation, the concentrations of the proteins were adjusted to 0.09 mg/ml and the CD spectra of the proteins were recorded at 25°C on a Chirascan (Applied Photophysics) CD spectrometer between 190–260 nm at medium scan speed, with 1 nm step length, in a cuvette of 1 mm path length. The final spectrum was an average of three repeat scans. Background corrections for buffer were done. The spectra for Als5 and Als5-SS were obtained by subtracting the spectrum of GST protein from the respective spectrum of GST-Als5 and GST-Als5-SS using the Pro-Data software that comes along with the instrument. The resultant spectra were further analysed using CONTIN [14].

### Adhesion Assay

Freshly purified GST-Als5 and GST-Als5-SS were checked for their ability to bind to human collagen type IV, following a slightly modified method to that reported [8]. Briefly, a 96-well flat bottom plate was coated with different amounts of collagen type IV and incubated overnight at 4°C. The plate was then washed thrice with PBS and twice with elution buffer. 200 µl of 0.09 mg/ml of either GST-Als5-SS, GST-Als5 or GST (control) was added to each well and incubated at 37°C for 1 hour. The wells were blocked for 1 hour with 5% skimmed milk in PBS and then washed 5 times with wash buffer (1% skimmed milk, 0.025% Tween-20 in PBS). Thereafter, 200 µl/well of primary anti-GST antibody (diluted 1∶1,000) was added. After incubation at 37°C for 1 hour, the wells were washed five times with wash buffer and incubated at 37°C with HRP-conjugated secondary antibody (200 µl/well; diluted 1∶20,000) for 1 hour. The wells were rinsed five times with wash buffer, 100 µl of freshly prepared tetramethylbenzidine solution was added to each well and incubated at 37°C for 1 hour. OD_650nm_ was monitored on a plate reader (Spectramax M2). Appropriate controls (without immobilised collagen, without GST-Als5/GST-Als5-SS proteins, and without primary antibody) were also done. “Buffer control” refers to the control with no collagen immobilized.

### Transmission Electron Microscopy (TEM)

TEM studies were carried out on a JEOL 2100F. The proteins (0.09 mg/ml) were incubated at 37^o^C for 2 weeks prior to the measurements. The proteins were spotted on a carbon-coated copper grid and positively stained with 2% uranyl acetate. The sample was then dried and examined by TEM using a 210 kV accelerating voltage.

### Peptide Binding Studies

Binding of GST-Als5 and GST-Als5-SS proteins to different peptides was monitored by fluorescence emission spectroscopy. Freshly purified samples of protein were used each time. 0.02 mg/ml of the proteins were taken and the tryptophan-specific emission spectra were monitored between 310 nm to 400 nm by excitation at 295 nm. The peptides were titrated into the protein solutions, incubated for 5 minutes after each addition, and emission spectra recorded. Both excitation and emission bandwidths were fixed at 5 nm and all spectra were averages of 5 scans. All spectra were corrected for buffer and peptide backgrounds. Purified GST alone at the same concentrations did not yield any significant fluorescence emission spectrum.

All binding data were analyzed using a one-site saturation model. Binding studies were done at different temperatures, and ΔG^o^ for the protein-peptide interactions calculated using the equation: ΔG^o^ =  –*RTlnK_a_*. ΔH^o^ and ΔS^o^ were obtained from the slope and intercept of the van’t Hoff plots according to the equation: 
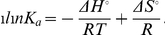


### Modelling and Docking Studies

The three dimensional structure of Als5 N-terminal domain (Als5Nt), Als5Nt in complex with portion of the C-terminal SS peptide (residues 1337–1347), Als5Nt with both the SS peptide and a peptide ligand (EHAHTPR) were modelled using the Rosetta 3.2 biomolecular comparative modelling and docking suite [15].

#### Model of Als5Nt

The structure of N-Terminal domain of the *C. albicans* Als9–2-apo form (PDB ID 2Y7N) was used for the construction of Als5Nt. The modelled protein shared 66% identity and 78% similarity with Als9–2.

#### Model of Als5Nt-SS peptide and Als5Nt-SS peptide in complex with peptide ligand

The structure of Als5Nt bound with a portion of the flexible SS peptide was obtained using Rosetta FlexPepDock module [16]. The starting complex structure was built on the basis of coarse-grained structural representation of the signal peptide and the receptor, as seen in the βG2′ strand in N-terminal domain of *C. albicans* Als9–2 in complex with human fibrinogen γ peptide (PDB ID 2Y7L). The last 11 amino acid residues (KFISVALFFFL) from the C-terminal signal sequence peptide of Als5-SS were modelled using 2Y7L C-terminal end which forms an extended strand (βG2′) over domain N1 in the Als9–2 structure [17]. Based on the docking simulation results 1000 models were generated. The ensembles were sorted by Rosetta scoring function, out of which the best model was selected for representation.

The model of Als5Nt-C-terminal signal peptide in complex with peptide ligand was then obtained using 2Y7L as template. The peptide ligand (EHAHTPR) was modelled using Fg-γ peptide as template.

All the structures were further minimized to eliminate bad atomic contacts. The molecular minimization simulations were done with the help of AMBER [18] molecular dynamics package using amber force field and steepest descent algorithm to remove close van der Waals contacts, followed by conjugate gradient minimization until the energy was stable in sequential repetitions. All hydrogen atoms were included in the calculation.

## Results

**Figure 1:**
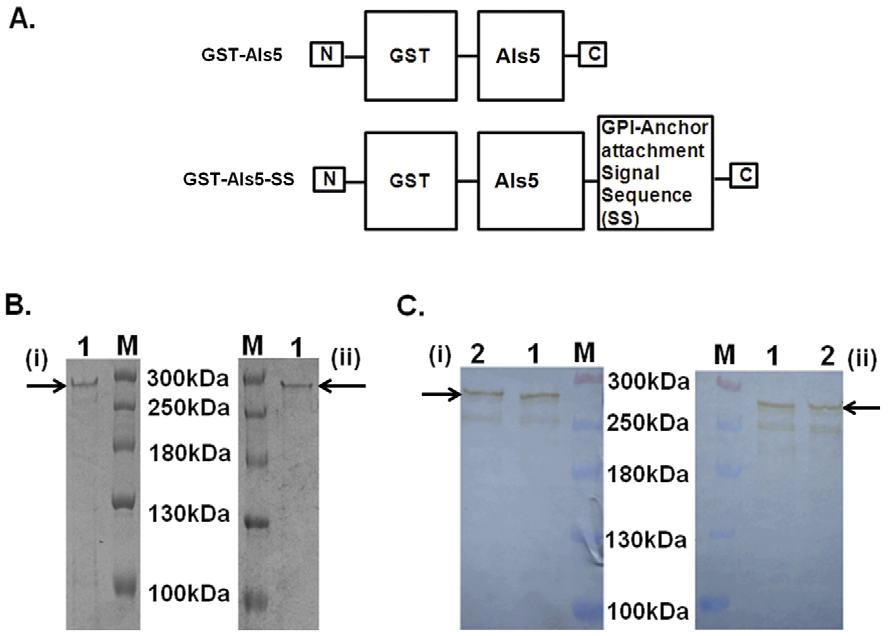
Preliminary characterization of GST-Als5 and GST-Als5-SS. (A) Cartoon depicting the difference between GST-Als5 and GST-Als5-SS. GST is fused at the N-terminus of both the proteins. GST-Als5 has only the protein sequence of the Als5 mature protein while GST-Als5-SS has, in addition to the Als5 mature protein sequence, the GPI-anchor attachment signal sequence (SS) of 20 amino acids also included in the expressed protein. (B) SDS-PAGE showing purification of GST-Als5 and GST-Als5-SS. The proteins were expressed and purified as described in Experimental Procedures. The purified elutes were run on a 6% SDS polyacrylamide gel and stained using Coomaassie Brilliant Blue R250. (i) Purification of GST-Als5. (ii) Purification of GST-Als5-SS. In each gel, Lanes M: Molecular weight markers; 1: elution fraction with 10 mM glutathione. The arrow indicates the bands of interest. The purified proteins showed a molecular weight much higher (around 270 kDa) than expected (around 168 kDa). The higher than expected molecular weights for both the proteins can be attributed to high content of serine and threonine residues in both the proteins [8]. (C) Western Blot confirmations of the purified GST-Als5 and GST-Als5-SS. The purified proteins were run on a 6% SDS polyacrylamide gel and transferred to PVDF membrane. The membrane was probed using anti-GST and anti-Als5 antibodies as described in Experimental Procedures. (i): Blot using anti-GST antibody. (ii): Blot using anti-Als5 antibody. Lanes M: Pre-stained protein molecular weight markers; 1: Purified GST-Als5; 2: GST-Als5-SS. The arrow indicates the bands of interest. Both the antibodies recognized the protein bands of around 270 kDa.

In order to address the issue of whether the SS is merely a signal for GPI anchor attachment in Als5 or whether it also has a say in the conformation and function of the protein, we generated the full length Als5 as well as its variant carrying the GPI-anchor signal sequence (Als5-SS) as GST-fusion products (Figure 1A) and did a comparative study of their conformation and function. The results are presented below.

### Cloning, Expression and Purification of GST-Als5 and GST-Als5-SS

The N-terminally GST-tagged protein variants, without the signal sequence (Als5) and with the GPI anchor attachment signal sequence (Als5-SS) were generated and confirmed as described in Methods (Figure S1). The proteins were expressed in *E. coli* BL21 strain. The prokaryotic host was chosen for the expression of the proteins so that the GST-Als5-SS protein would be obtained with the SS peptide intact. A eukaryotic host would have resulted in processing of the GST-Als5-SS, and would not have allowed us to address the role of the SS sequence in the conformation/function of Als5.

The molecular weight of the GST tag is ∼26 kDa. Hence, for the GST-Als5 and the GST-Als5-SS proteins the molecular weights were expected to be around 166 kDa and 168 kDa, respectively. However, we observed bands of M_r_ ∼270 kDa, for both GST-Als5 and GST-Als5-SS (Figure 1B). This marked difference between the observed molecular weights in SDS-PAGE and the expected molecular weights has been previously attributed to the high content of hydroxyl amino acids in proteins [11]; both Als5 and Als5-SS are rich in serine and threonine residues. The concentrations of the proteins are low, as can be seen from the gels. Attempts to concentrate the proteins any further, however, resulted in most of the GST-Als5 protein aggregating and precipitating out.

Along with the ∼270 kDa main band, a much fainter lower band is also present in both gels despite the use of protease inhibitor cocktail in the lysis buffers which is not so obvious when viewed by Coomassie Brilliant Blue R250 staining (panels (i) & (ii) of Figure 1B) but shows-up as a faint band on Western Blots using anti-GST (Figure 1C, panel (i)) as well as anti-Als5 antibodies (Figure 1C, panel (ii)). The levels of the degradation products were roughly similar in the two cases. That these were GST-tagged proteins and probably represent degradations of the main proteins of interest was obvious from the Western Blots. From the intensities of the bands, we assessed that the levels of purity of the two proteins were > 90% in the elution samples and thus the levels of the degradation products were not likely to significantly alter the results of our experiments.

The identities of the two proteins were confirmed by peptide mass fingerprint using MALDI-TOF analysis as well as ESI-MS. The data obtained for GST-Als5-SS using MALDI-TOF, ESI-MS and intact mass analysis (168.6 kDa) is given in Figures S2, S3 and S4, respectively.

It must be pointed out that a PreScission^TM^ protease site exists between the fusion tag and the Als5 proteins in the two constructs. However, our attempts to cleave off the fusion tag with the protease met with poor success. The PreScission^TM^ protease is also available as a GST-tagged enzyme and it is possible that steric factors precluded the interaction of the protease with the cleavage site in our fusion protein. Additionally, our protein yields to begin with were very low (∼0.09 mg/ml) and this too could have affected the efficiency of the cleavage. Concentrating the proteins was not an option since GST-Als5 tended to aggregate and precipitate out of solution. Hence all our studies were carried out with the fusion proteins. We used similarly expressed and purified GST as the control for all our experiments. Similar studies with GST-tagged proteins, including conformational studies, have been reported by other groups previously (*cf.* [19]).

**Figure 2:**
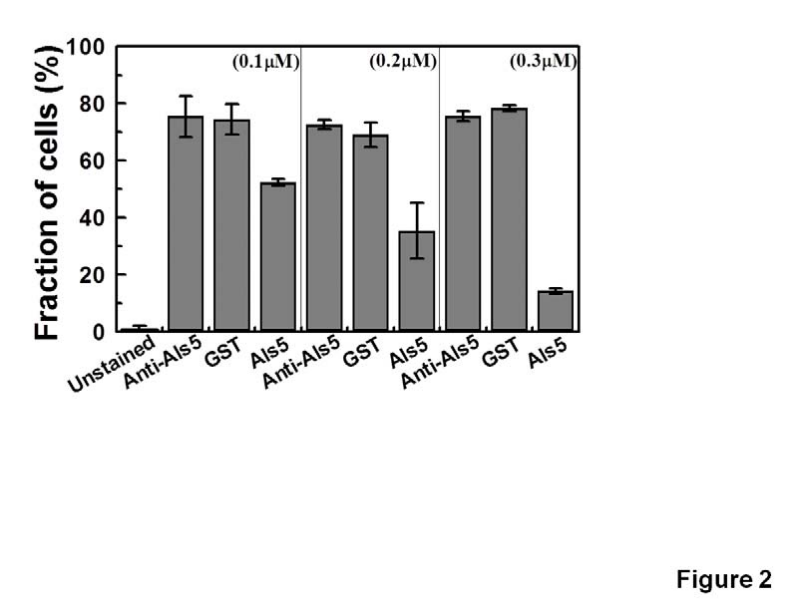
GST-Als5 specifically blocks binding of anti-Als5 antibody on Candida cell surface. Candida SC5314 cells were grown to an OD_600nm_ of 0.5. 500 µl of the cells after pelleting down were washed with PBS and then incubated with anti-Als5 antibody for 1 hour. This primary antibody was detected by a secondary antibody that was conjugated with TRITC and detected using flow cytometry as described in the text. The y-axis in the figure represents the percent fraction of fluorescently labelled cells. Unstained: Cells were incubated with the primary Ab buffer (instead of anti-Als5 Ab), washed thrice with PBS and then incubated with the fluorescently labelled secondary antibody before being detected using flow cytometry. Anti-Als5: Cells were incubated with anti-Als5 primary Ab, washed thrice with PBS and then incubated with the fluorescently labelled secondary antibody before being detected using flow cytometry. GST: Cells were incubated with GST, then with anti-Als5 Ab, washed thrice with PBS, and then incubated with the fluorescently labelled secondary antibody before being detected using flow cytometry. Als5: Cells were incubated with GST-Als5, then with anti-Als5 Ab, washed thrice with PBS, and then incubated with the fluorescently labelled secondary antibody before being detected using flow cytometry. All incubation steps were carried out at 37^o^C for 1 hour. Concentrations of the GST as well as GST-Als5 used in the competition assays are as shown in the figure. The data presented is mean of 3 independent experiments done in duplicates. The anti-Als5 antibody generated in this study was able to recognize and bind to the adhesins on Candida cell surface, as exhibited by the fraction of fluorescent cells detected using TRITC labelled secondary Ab. The presence of GST-Als5, but not GST, inhibited the binding of anti-Als5 antibody to *C.albicans* cell surface in a concentration dependent manner, thus demonstrating the specificity of the interaction.

In order to confirm the identity of the Als5 protein, we also designed the following experiment. We first used anti-Als5 antibodies to bind to *C. albicans* SC5314 cell surface. Using a TRITC labelled secondary antibody, we detected the bound primary antibodies on the Candida cells by flow cytometry. As can be seen from Figure 2, a significant fraction of the cells were bound by the antibodies. Next, we attempted to compete out the binding of the anti-Als5 antibodies to the *C. albicans* cells using GST-Als5. To ascertain the specificity of the interaction, we used both GST as well as GST-Als5 in these competition assays, where the individual proteins were present in the sample before addition of the primary antibodies. As can be seen from Figure 2, while GST could not inhibit the binding of the antibodies on the cell surface, the GST-Als5 protein could inhibit the interaction of the primary antibodies to the cell surface in a concentration dependent manner.

### Als5 Binds Collagen Type IV

**Figure 3:**
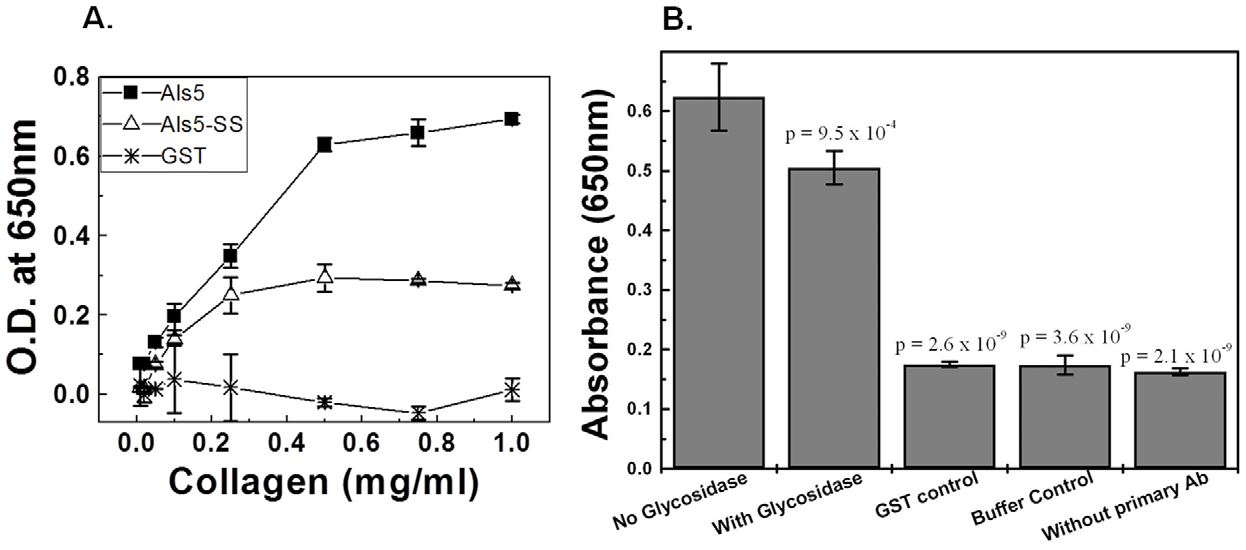
Adhesion of Als5 to collagen type IV involves recognition of carbohydrate residues on collagen type IV but is not solely determined by it. (A) Adhesion of GST-Als5, GST-Als5-SS to collagen type IV. Different amounts of collagen type IV, as shown in the figure, were immobilized in 96-well flat bottomed ELISA plates and incubated with the proteins (0.09 mg/ml) of interest at 37^o^C for 1 hour. The adhesion assay was carried out as described in Experimental Procedures. Adhesion of the proteins to collagen type IV was correlated to the absorbance at 650 nm observed by the oxidation of TMB, the substrate of HRP. Filled square: binding of GST-Als5; open triangle: binding of GST-Als5-SS; star: binding of GST alone. The binding by GST alone represents the non-specific binding. Buffer controls (OD 650_nm_ of 0.126 ± 0.003), corresponding to no immobilized collagen, have been subtracted in all cases. Signal from the controls (without immobilised collagen, without GST-Als5/GST-Als5-SS/GST proteins, and without primary antibody) were similar to the buffer control. The data presented is mean of 3 independent experiments done in duplicates. GST-Als5 showed greater adherence to collagen type IV as compared to GST-Als5-SS. GST showed very poor interaction with collagen type IV. (B) Treatment of collagen type IV with N-Glycosidase F results in decrease in adherence of GST-Als5. Collagen type IV (100 µl; 1 mg/ml) was immobilised overnight at 4^o^C in 96-well ELISA plate. The wells were then rinsed 5 times with PBS and incubated with N-glycosidase F (Roche) for 2 hours at 37^o^C in 50 mM sodium phosphate buffer (pH 8.0) containing 25 mM EDTA and 1% v/v β-mercaptoethanol. After washing 5 times with PBS, GST-Als5 was added and incubated for an hour. Adherence to collagen type IV was detected as already described. No Glycosidase: Only the buffer of the glycosidase was added. With glycosidase: 0.5 U glycosidase was added. All other wells were also incubated at 37^o^C with buffer of the glycosidase. GST control: GST was added in place of GST-Als5 to monitor the non-specific interaction; Buffer control: No immobilized collagen; Without primary Ab: The step where primary Ab was to be added was replaced with incubation with the buffer only. Average values for 3 independent experiments done in duplicates are shown with standard deviations. p-values representing statistical significance of data with respect to ‘No Glycosidase’ data are shown in figure. Flow chart representing the various steps of the assay is shown in Figure S5.

Several studies have shown that collagen type IV is one the proteins of the extracellular matrix that is specifically recognized by *C. albicans* cells during infection [9]. Homology studies identified Als5 as a protein homologous to collagen binding proteins [10]. We performed an adhesion assay that involved different amounts of immobilised collagen type IV as described in Methods. We observed interaction of each of the Als5 protein variants with collagen type IV which depended upon the amount of collagen used for the immobilization (Figure 3A). More interestingly, GST-Als5 had greater adherence to collagen type IV as compared to GST-Als5-SS. In contrast, GST alone showed very poor interaction with collagen type IV.

In order to see whether the carbohydrate chains on collagen type IV had any role in Als5 adhesion, we treated the immobilized collagen type IV in our adhesion assays with 0.5 U of N-glycosidase F (Roche). We observed a roughly 20% drop in adhesion of GST-Als5 to collagen type IV after deglycosylation with the enzyme for 2 hours (Figure 3B; flow chart of methodology in Figure S5). Increasing the deglycosylation time to 3 hours did not further reduce the adhesion of GST-Als5 to collagen type IV (data not shown). Thus, Als5 may use the carbohydrate side chains of collagen in addition to the peptide backbone of collagen and other similar proteins for adhesion.

That some adhesins may also use carbohydrate ligands present on collagen type IV was also suggested by Timoneda’s group [20]. More recently, the N-terminal domain of Als1 was shown to bind fucose-containing sugars from a glycan array with millimolar affinity, although the glycan could not significantly and specifically inhibit the binding of the adhesin to laminin or fibronectin [21].

### Secondary Structure of Als5 is Different from that of Als5-SS

To understand whether this difference in collagen-binding was due to conformational differences between the two proteins, we analyzed the structure of the two proteins using CD spectroscopy.

**Figure 4:**
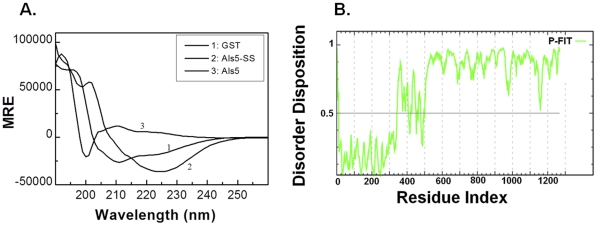
Secondary structure of Als5 and Als5-SS are perceptibly different. **(A)** **CD spectra of Als5 and Als5-SS.** Individual CD spectra were recorded for 0.09 mg/ml each of GST-Als5, GST-Als5-SS and GST and the spectrum for GST was subtracted from that of the GST-tagged fusion proteins in order to obtain the final CD spectra of Als5 (trace 3) and Als5-SS (trace 2). The spectrum of GST alone (1) is also shown in the figure. The values are reported as mean residue ellipticity (MRE) in degrees-cm^2^dmol^-1^. The curves were smoothened using the Savitzky-Golay smoothing function in the Origin software (Microcal). The spectrums of Als5 and Als5-SS are clearly distinct from each other. (B) Prediction of disordered regions in Als5 using PONDR-Fit. The Als5 protein sequence was fed into the on-line prediction program available at http://www.disprot.org/pondr-fit.php to predict whether there were any disordered regions in the protein. The grey line at 0.5 of Y-axis is the threshold; residues with score above 0.5 are predicted to be disordered while those below 0.5 are predicted to be ordered.

The CD spectra of Als5 and Als5-SS (after subtracting the spectrum of GST from that of the fusion proteins) are shown in Figure 4A. The structural differences between the two proteins are very obvious from the figure.

The Als5 spectrum closely resembled that of intrinsic pre-molten globules [22]. However, no prediction programs are currently available that have a database of CD spectra from intrinsically disordered proteins that would have enabled us to fit the CD spectrum of Als5 to such a model. So we chose to instead use an on-line prediction program, PONDR-Fit [23], to determine whether there are intrinsically disordered regions in the Als5 protein. As can be seen in Figure 4B, a large portion of the protein (∼70%), in its C-terminal half, is predicted to be significantly disordered. There is increasing evidence to suggest that such intrinsic disorder is very important for the functionality of many proteins, and disordered regions of many proteins are shown to attain structure only in the presence of ligand and/or during ‘function’ [22]. Given that the C-terminal domain is capable of mediating cell-cell adhesion [11], it is possible that the stalk region of these proteins remains flexible and attainment of structure in this domain is dependent on cell-to-cell contacts.

When analysed by CONTIN [14], Als5, has a β-strand-rich structure with a significant amount of β-turns and disordered regions (the closest matching solution suggested 25.4% β strand, 9.2% α-helix, 43.8% turn and 21.6% disordered regions while the average of all matching solutions suggested 38.3% β strand, 6.4% α-helix, 42.6% turn and 12.6% disordered regions) (Table S2). The structure in the CD signal is, perhaps, largely contributed by the N-terminal half of the protein which is predicted to be well-folded by PONDR-Fit.

There is experimental evidence to suggest that the N-terminal domain of Als5 and other Als-like adhesins may be well folded. The isolated N-terminal domain of Als5 has been estimated to contain 50.1% β-sheet, 26.9% disordered regions, 19.3% turns and only 3.7% α-helix using CD spectroscopy [10]. The NMR structure of the N-terminal domain of Als1 [24] as well as the crystal structure of the N-terminal domain of Als9 suggest that these adhesins are rich in β-strand content with a significant amount of flexible regions [17]. The isolated tandem repeat (TR) sequences of Als5 and other Als-like adhesins, folded into β-sheet rich structures when modelled using either Rosetta or LINUS [25]. Additionally, a 36-mer unglycosylated synthetic peptide from this region as well as highly glycosylated truncated mutant of Als5 containing the TR region appeared to have predominantly β-sheet rich architectures [25].

It is noteworthy, that the Als5-SS has a strikingly different conformation from that of Als5. Analysis for its secondary structure content using CONTIN revealed that Als5-SS, was predominantly α-helical (the closest matching solution suggested 73.6% α-helices and 26.4% disordered regions while the average of all matching solutions estimated 60.2% α-helices, 16.3% β strands, and 23.5% disordered regions) (Table S2).

### Als5 has a Greater Tendency for Aggregation than Als5-SS

**Figure 5:**
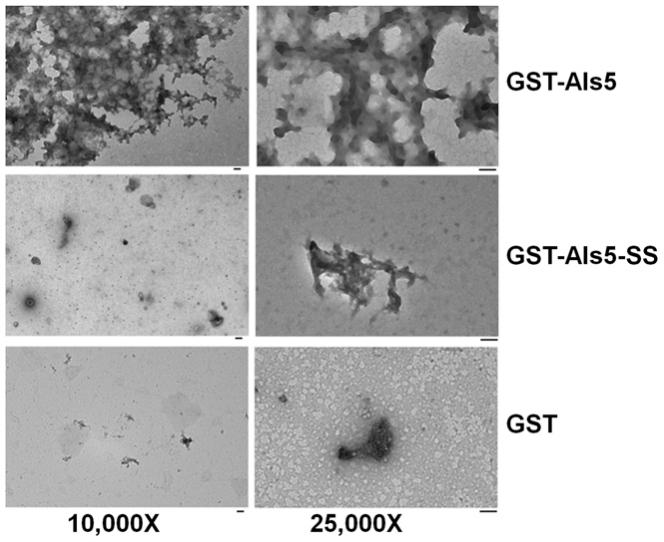
Transmission electron microscope images showing aggregation by GST-Als5. The proteins, GST, GST-Als5 and GST-Als5-SS (0.4 µM of each) were incubated at 37^o^C for 2 weeks before being examined under a transmission electron microscope. As can be clearly seen, GST-Als5, but not GST showed significant amount of aggregation, typical of β-sheet rich proteins. GST-Als5-SS, on the other hand, showed very poor aggregation under similar conditions. The scale bar in each panel represents 100 nm. Left panel: 10,000X magnification. Right panel: 25,000X magnification.

The higher β-sheet content of Als5 should also be reflected in a greater tendency for aggregation by the protein as compared to Als5-SS. In order to test this hypothesis, we incubated the protein at 37^o^C for 2 weeks and obtained TEM images for the proteins. As can be seen from Figure 5, GST-Als5 shows significantly higher amount of aggregation as compared to either GST-Als5-SS or GST alone. The aggregation of GST-Als5 that we observed is well in keeping with the study by Ramsook *et al.* who showed that Als5 tended to form amyloids and precipitate out of the solution into the medium, when expressed in *S. cerevisiae* [13].

**Figure 6:**
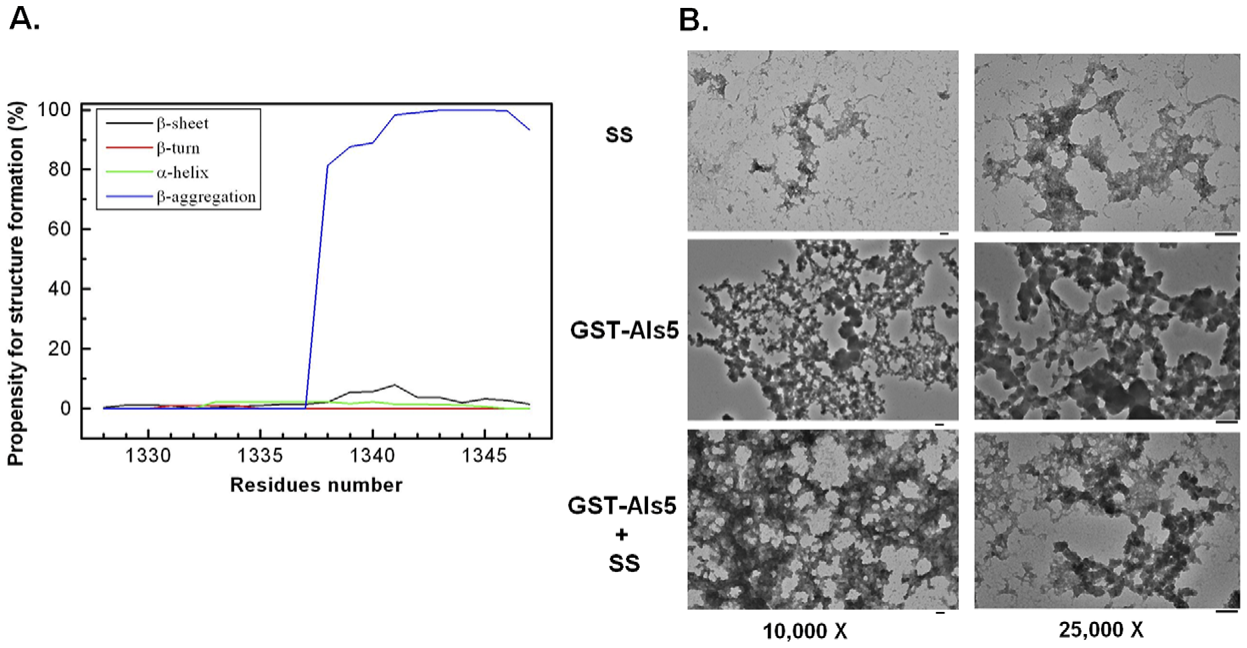
β-aggregation is exhibited by SS both in the absence and presence of GST-Als5. (A) Prediction of β-aggregation potential for the GPI anchor attachment signal sequence (SS). The prediction of β-aggregation potential for the SS was done using TANGO (residues 1328–1347). Also shown is the propensity for α-helix, β-turn and β-sheet formation in the unfolded polypeptide chain as predicted by TANGO. As can be seen from the figure, the C-terminal half of the SS has a high propensity for β-aggregation. (B). Transmission electron microscope images showing aggregation by SS and SS in the presence of GST-Als5. The samples, SS (40.0 µM), GST-Als5 (0.4 µM) and GST-Als5 (0.4 µM) + SS (40.0 µM) were incubated as 37^o^C for two days before TEM images were recorded. As can be seen from the figure, the SS alone is capable of aggregation. GST-Als5 aggregates both in the absence as well as in the presence of SS. In contrast, in Figure 5 we showed that even after two weeks of incubation GST-Als5-SS showed no propensity for aggregation, clearly indicating that the conformation adopted by the two Als5 variants is different.

Why does the Als5-SS not aggregate? The amyloidogenic region that was previously identified in the Als5 sequence [12] also exists in Als5-SS. Additionally, prediction by TANGO [26]–[28] suggests that the C-terminal half of the SS also has a very high propensity for beta-aggregation (Figure 6A). TEM studies confirm the potential of the SS peptide to form aggregates (Figure 6B). Had the amyloidogenic and SS sequences been exposed to solvent, this should have resulted in a high tendency for aggregation in Als5-SS. We tested this hypothesis by simultaneously incubating GST-Als5 and the SS peptide at 37^o^C for 2 days. We observed that SS peptide as well as the GST-Als5 sample containing the SS peptide showed significant amount of aggregation (Figure 6B). Clearly, the Als5-SS does not have its amyloidogenic regions exposed for beta aggregation and thus has a conformation that differs from that of Als5.

We looked for clues from the available crystal structure to explain how Als5-SS could differ in conformation from Als5 despite differing in only 20 residues at the C-terminus. One probable hypothesis is that the C-terminal SS is able to fold back and fit either into the peptide-binding pocket of the protein or dock over the N1 domain as has been reported for the N-terminal domains of both Als9 and Als1 [17]. If the SS in Als5-SS folds back to interact with the N-terminal half of the protein, it is possible that it would also introduce torsion into the protein chain, keeping it perhaps in a more structured conformation and forcing the C-terminal domain into a predominantly α-helical arrangement. This could also perhaps lead to shielding/burial of the amyloidogenic region (residues 325–329; Figure S6) of the protein. The cleavage of the SS peptide would result in release of this torsional constraint, exposing its amyloidogenic region, while simultaneously allowing the C-terminal domain of the protein to adopt a more relaxed conformation.

**Figure 7:**
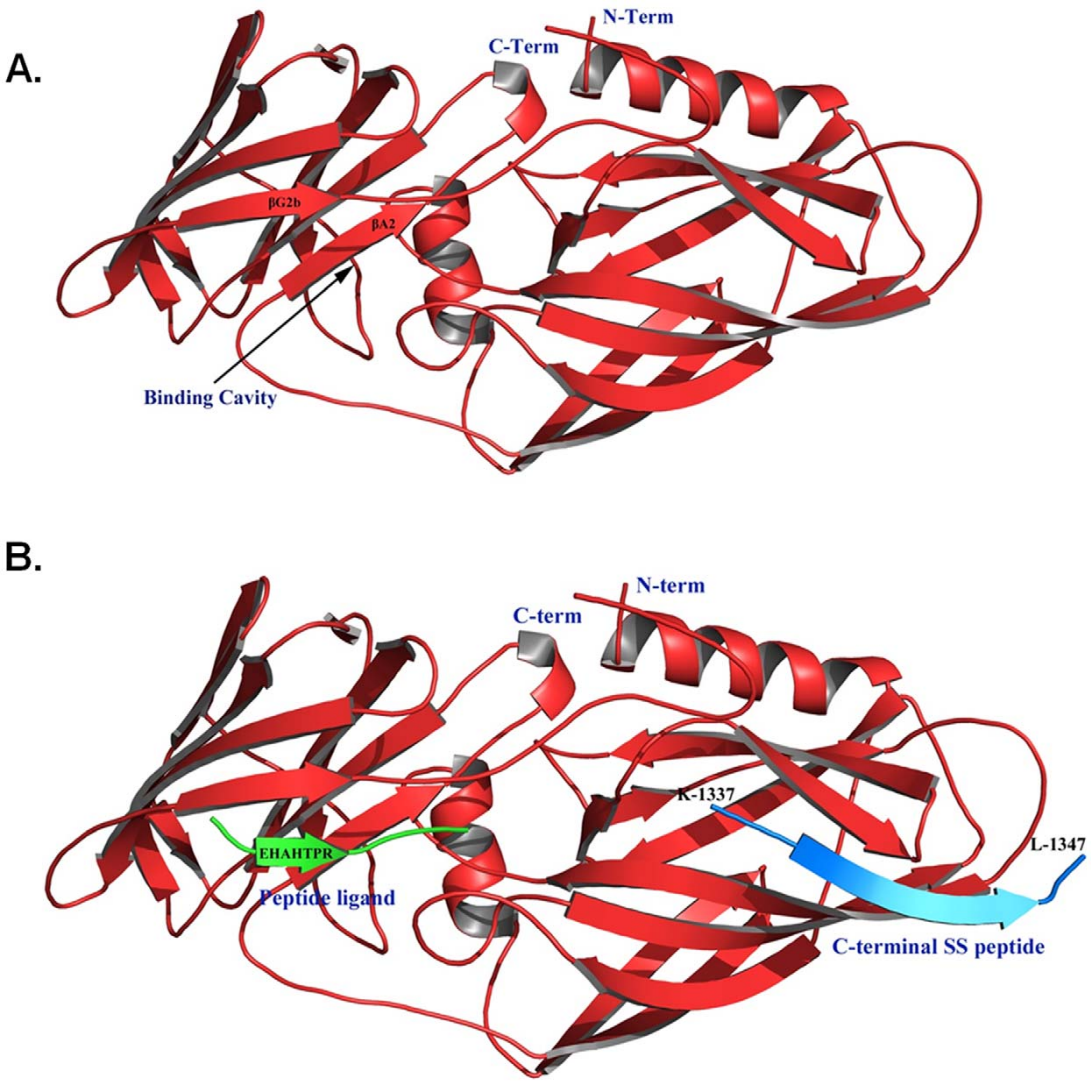
Cartoon representation of Rosetta models for Als5Nt complexes. (A) Low energy Rosetta model of Als5Nt. (B) Low energy Rosetta docked model of Als5Nt bound to peptide ligand (EHAHTPR) as well as to the eleven-residue long C-terminal peptide (KFISVALFFFL) from SS. The Als5Nt is colored in red, the peptide ligand is shown in green and the SS peptide is shown in blue. The figures were generated using the program PyMOL.

We hypothesised that the SS might fold back and interact with the N1 domain (nomenclature as per [17]) rather than the peptide binding pocket of Als5-SS because the ligand binding pocket of Als5-SS binds peptide ligands and is therefore not likely to be occupied by the SS (from homology modelling, the peptide binding pocket in an energy minimised model of N-terminal domain of Als5 does not appear to be capable of accommodating more than one peptide; Figure S7A). To test this hypothesis, we performed homology modelling of the N-terminal domain of Als5 (Als5Nt) along with the SS peptide as described in Methods. The energy minimised model of Als5Nt is shown in Figure 7A. We observed that the C-terminal half of the SS peptide can readily replace the C-terminal end which forms an extended strand (βG2′) over domain N1 in the Als9 structure both in the presence (Figure 7B) or absence (Figure S7B and S7C) of the peptide ligand (EHAHTPR).

While we are limited by the availability of structural data for the full-length protein or even the C-terminal domain of this protein, such a mechanism would best explain our experimental results.

### Specificity of Ligand-binding of the Two Proteins is also Different

In order to assess whether the ligand binding pockets of the two proteins could be correlated to the differences that we observed in their global conformation and in the differences in their adhesion to collagen type IV, we set out to map the interaction of the two proteins with a set of specific peptide ligands that have been previously shown to be ligands for Als5 amplified from a human-isolated CA1 strain of *C. albicans* and heterologously expressed on the cell surface of *S. cerevisiae* [29].

We generated four peptides, KLRIPSV, AYKSLMT, EHAHTPR and VSPIRLK. The first two peptides were chosen because they had been shown to be specific for Als5 and the third because it was reported to be incapable of binding to Als5 [29]. We chose, additionally, to make a fourth peptide with the reverse sequence of KLRIPSV in order to determine specificity of the interaction.

We observed that the intrinsic tryptophan fluorescence of the protein was sensitive to peptide binding and could be used as a reporter for the interaction. Als5 and Als5-SS have 13 and 14 tryptophan residues, respectively, of which 13 are present in the N-terminal half of the proteins where the ligand is expected to bind. The crystal structure of N-terminal domain of Als9 bound to peptide also suggests a role for a conserved tryptophan in the ligand binding [17].

The change in fluorescence emission intensity upon titration with the peptide ligands was monitored (see Figures S8A, S8C, S8E, S8G and Figures S9A, S9C, S9E, S9G, respectively) and the binding plots obtained (Figures S8B, S8D, S8F, S8H and Figures S9B, S9D, S9F, S9H, respectively) were used to calculate dissociation constants (K_d_) for the interaction (Table 1).

**Table 1:** Thermodynamic parameters for peptide binding to GST-Als5 and GST-Als5-SS.

Peptide	Protein	K_d_ × 10–^8^						ΔG^o^						ΔH^o^	ΔS^o^
		M						kJ.mol^-1^						kJ.mol^-1^	kJ.mol^-1^.K^-1^
		15^o^C	27^o^C	37^o^C	42^o^C	47^o^C	52^o^C*	15^o^C	27^o^C	37^o^C	42^o^C	47^o^C	52^o^C*		
KLRIPSV	Als5	N.B.	N.B.	3.4±1.2	0.4±0.2	0.7±0.3	0.7±0.2	-	-	-43.91	-50.36	-50.39	-50.85	94.3	0.45
	Als5-SS	N.B.	5.3±0.6	1.7±0.5	-	1.4±0.2	0.5±0.07	-	-41.85	-46.21	-	-48.12	-51.42	64.21	0.35
AYKSLMT	Als5	N.B.	N.B.	1.3±0.7	0.9±0.4	1.3±0.6	1.2±0.7	-	-	-47.19	-48.68	-48.63	-49.77	-3.36	0.14
	Als5-SS	N.B.	7.7±0.2	0.6±0.02	-	0.9±0.2	0.2±0.07	-	-40.9	-49.04	-	-46.45	-53.57	77.75	0.4
EHAHTPR	Als5	N.B.	N.B.	16.0±3.8	3.4±0.8	3.4±1.1	1.1±0.4	-	-	-40.46	-45.09	-45.83	-49.63	139.87	0.58
	Als5-SS	6.5±1.3	8.2±1.6	4.4±0.4	-	2.5±0.9	0.8±0.2	-39.65	-40.73	-43.71	-	-46.71	-50.25	34.12	0.25
VSPIRLK	Als5	-	N.B.	-	-	N.B.		-	-	-	-	-		-	-
	Als5-SS	-	N.B.	-	-	N.B.		-	-	-	-	-		-	-

Peptides ligands used are from a previously identified ligand library [29].

**Figure 8:**
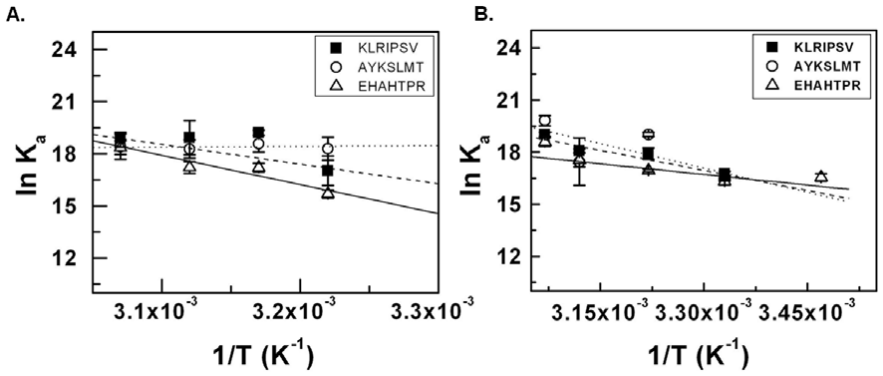
van’t Hoff plots of peptide-binding by the proteins. (A) Binding of GST- Als5 with different peptides. (B) Binding of GST-Als5-SS with different peptides. K_a_ refers to the association constants for the protein-peptide interactions.

The dissociation constants (K_d_) for peptide binding to GST-Als5 and GST-Als5-SS were obtained from the binding plots shown in Figure S8 (B, D, F & H) and Figure S9 (B, D, F & H) respectively. The standard state free energy (ΔG^o^) values at different temperatures were calculated using the equation ΔG^o^ =  -RTln K_a_ (where K_a_ refers to the association constant for the protein-peptide interaction). Standard state enthalpy (ΔH^o^) and entropy (ΔS^o^) values were obtained from the van’t Hoff plots for the protein-peptide interactions (Figure 8A & 8B). N.B. implies no binding observed.

* The secondary structure of the Als5 and Als5-SS were mildly perturbed at 52^o^C as compared to that at the lower temperatures from CD spectral studies (data not shown).

We discovered that neither of the proteins bound any of the peptides at 4^o^C. However, at 15^o^C, GST-Als5-SS bound EHAHTPR with K_d_ approximately 65 nM but did not interact with either KLRIPSV or AYKSLMT at this temperature. GST-Als5, on the other hand showed no binding with any of the peptides under similar conditions. At 27^o^C, GST-Als5-SS bound to all three peptides with roughly comparable affinities. Under these conditions GST-Als5 did not bind any of the peptides. At 37^o^C, 47^o^C and 52^o^C both proteins showed significant amount of binding to all the three peptides.

The scrambled peptide VSPIRLK, on the other hand, showed no affinity for either of the protein variants at 27^o^C and 47^o^C, suggesting that the interaction of the proteins with the other three peptides was specific.

Notably, while GST-Als5-SS binds to the peptides at lower temperatures, GST-Als5, with the GPI anchor attachment signal removed, begins to recognize these peptides at higher temperatures only, clearly indicating that the functionalities, and therefore conformations, of the two protein variants are different. That both proteins bind EHAHTPR, a peptide previously reported to not bind Als5 [29], suggests that strain-specific variations or allelic differences could result in subtle differences in the specificities of Als5 proteins expressed on the cell surface of *Candida albicans*. Such strain-specific and allelic variability in Als proteins is well documented [30].

We concluded from these studies that the two proteins had some differences in the ligand-binding pocket that manifested in differences in peptide-recognition by the proteins. It is possible that the interaction of the SS with the N-terminal domain of the protein in Als5-SS, as suggested in the above model, perturbs the ligand binding pocket of the protein and manifests in these differences in ligand binding that we observe.

The high affinity of the interaction of Als5 for its peptide ligands is also interesting to note in the biological context. Adhesion of the pathogen to host surfaces could be dictated by very low concentrations of appropriate ligands on the cell surface. Similar high affinity interactions between adhesins and their ligands have been previously reported. Recently Salgado *et al*. estimated that the isolated N-terminal domain of Als1 bound to its peptide ligands with affinities better than 10 µM [17] and Donohue *et al.* showed by SPR studies that the affinity of the N-terminal domain of Als1 for fibronectin and laminin was in the low micromolar range [21].

Using the binding data, we also obtained van’t Hoff plots for the interaction of the two proteins with the peptides (Figure 8A and Figure 8B, respectively) and calculated thermodynamic parameters for the interaction (Table 1).

As can be seen from Table 1, the interactions of the two proteins with the peptides are largely endothermic processes. Further, the binding affinity of the peptides to the proteins improves with temperature, suggesting that the interactions are predominantly hydrophobic in nature and are primarily driven by entropic considerations. This is also supported by the increasingly negative values of free energies for the interaction with increasing temperature. Recent crystal structure information on the peptide binding pocket of Als9 also supports this inference; a set of hydrophobic residues in the binding pocket appear to make extensive contacts with the bound peptide [17].

## Discussion

In order to understand the effect of an uncleaved GPI anchor attachment signal sequence on the conformation and function of a protein to be GPI-anchored, we chose to study the Als5 protein of *Candida albicans*.

The first requirement of this study was to be able to show that the Als5 protein expressed and purified from the bacterial cells was indeed ‘functional’.

*In vitro* studies on the conformation and function of Als proteins have been challenging, not least due to the size of these proteins. To date, no detailed *in vitro* characterization of a full-length adhesin has been reported. But clues on the function and properties of Als5 can be inferred from other available studies. Studies using heterologously expressed Als5 on the cell surface of *S. cerevisiae* showed that Als5 could induce adherence of the host cells to collagen type IV, fibronectin and other extracellular matrix proteins as well as to epithelial and endothelial cells [9] and the process was primarily dictated by hydrophobicity [31]. Further, yeast cells expressing Als5 could cause co-adhesion with bacterial cells [32].

Our current set of studies, using purified GST-Als5, demonstrated that the recombinant full-length protein is capable of mediating adhesion. The protein adhered to collagen type IV, a protein of the basal lamina. Additionally, we show that carbohydrates on collagen type IV also participate in the adhesion, although, they do not appear to be the sole determinants of the interaction.

Our studies also indicated that the adhesion mediated by Als5 is predominantly driven by entropic considerations and therefore is determined primarily by hydrophobic interactions. This too is well in keeping with available literature [31] and with the recent report on the crystal structure of the N-terminal domain of Als9, another *C. albicans* adhesin, which showed a set of hydrophobic amino acids participating in ligand binding [17].

Since this protein is expressed in *E. coli* and is therefore not glycosylated, it would seem that glycosylation of Als5 *per se* may not be an essential condition for adhesion mediated by this protein. This may not be surprising, given that the adhesion is expected to be mediated by the N-terminal domain which is predicted to be poorly glycosylated [4]. It remains to be seen, however, whether a glycosylated variant of this protein would show better adhesion *in vitro* as compared to this non-glycosylated form.

Previous reports with shorter fragments of Als5, deletion constructs containing only the conserved immunoglobulin and Thr-rich domains (Ig-T) as well as the one which additionally possessed six tandem repeat domains (Ig-T-TR_6_), suggested that the functional domains of Als5 were predominantly β-sheet-rich [11]. In a recent paper, Ramsook *et al.* [13] showed that soluble GPI-less Als5 when expressed in yeast tended to rapidly form precipitates with amyloid characteristics, a feature typical of β-sheet aggregation. Thus, the functional Als5 is expected to be β-sheet-rich and aggregation-prone.

Our results regarding the secondary structure and aggregation status of Als5 are well in keeping with available literature and confirm that this variant embodies the final functional form of the protein.

Next, we looked to understand how the conformation and function of Als5-SS, carrying the C-terminal signal sequence for GPI anchor attachment, compared with that of Als5. We found that in contrast to Als5, Als5-SS was predominantly α-helical in nature and exhibited a much lower tendency to form aggregates. The presence of the SS at the C-terminus also attenuates the affinity of the peptide binding pocket of Als5 for its specific peptide ligands, indicating that not only is the global conformation altered, but the local conformation of the peptide-binding pocket, located at the N-terminal half, is also perturbed. Additionally, it adheres lesser to collagen type IV in comparison to Als5. The reduced self-aggregation as well as adherence to collagen type IV reflects the conformational difference of this precursor form of the protein from that of the mature Als5. How does the short C-terminal signal sequence impart such a large conformational difference between Als5-SS and Als5? We favour a model wherein the SS folds back to interact with the N-terminal domain of the protein in Als5-SS and in doing so compels the C-terminal domain of the protein to also adopt a more structured conformation. The removal of the SS lifts the torsional constraint imposed upon the C-terminal domain, allowing it to assume a more relaxed, disordered conformation in Als5.

As mentioned earlier, proteins meant to be GPI-anchored, are completely translocated into the ER lumen with the C-terminal signal sequence intact [3]. This is subsequently and rapidly acted upon by the GPI-transamidase complex, resulting in cleavage of the SS and its replacement by a pre-formed GPI anchor precursor. Thus, for a brief while, before the GPI-transamidase complex acts, the precursor form of the GPI-anchored protein is present in the lumen of the ER and capable of interacting with other proteins in its vicinity under normal conditions.

However, under a number of conditions, the concentration of this transient species can build up in the ER. For instance, any aberrations in the GPI anchor biosynthetic pathway can reduce the levels of pre-formed GPI anchor available and result in reduced GPI anchoring of proteins. Paroxysmal nocturnal hemoglobinuria (PNH) and inherited GPI deficiency (IGD) are two examples of problems associated with mutations in crucial steps of the GPI biosynthetic pathway in humans [33]. Mutations in the C-terminal signal sequence of a protein to be GPI anchored can also result in its reduced GPI anchoring. When working with over-expression systems involving GPI anchored proteins too, the possibility of these precursor proteins accumulating within the ER lumen is real.

How does the cell deal with the precursor proteins in such conditions? The mechanisms employed are likely to be different depending on the cell type as well as the nature of the protein. It has been shown, for example, in neutrophils of PNH patients that precursor GPI-anchored proteins accumulate in the Golgi [34]. In GPI-deficient LM-TK-mouse fibroblast cells, the precursor form of placental alkaline phosphatase is transported out, rapidly inactivated, and degraded by the lysosomal compartment [35].

With proteins like Als5, which have a high propensity to form aggregates, minimizing unwanted interactions in the crowded environment of the ER lumen, would be quite a challenge even under normal conditions. This problem would be more acute under abnormal GPI anchoring conditions. Our results suggest that the precursor form of Als5 is likely to be folded into a predominantly α-helical protein before removal of the signal sequence. The cleavage of the C-terminal SS converts it to the mature β-strand-rich form of the protein. Thus, it would appear that the C-terminal signal sequence not only directs the attachment of the GPI anchor but also holds the protein in an α-helical conformation that is less likely to be aggregation-prone in the absence of the SS removal. It is possible that similar strategies are employed in the case of precursor forms of other GPI-anchored enzymes and proteins, including adhesins, in order to “control” the function of these proteins during their transit through the ER.

## Supporting Information

Figure S1 **Cloning of** ***ALS5*** **and** ***ALS5-SS*** **regions in pGEX-6-P2 vector. (A)** Cloning of *ALS5* region in pGEX-6P-2 vector. *ALS5* sequence was amplified by PCR (Table S1) and the amplicon was digested with BamHI and XhoI restriction enzymes. The restriction enzyme digested amplicon was ligated into similarly digested pGEX-6-P2 vector and the construct after ligation was confirmed by restriction enzyme digestion. Lane 1: Uncut plasmid after ligation of *ALS5* gene in pGEX-6P-2 vector, Lane M: DNA molecular size marker, Lane 2: pGEX-6P-2 vector with *ALS5* region cloned and restricted with BamHI and XhoI enzymes, resulting in release of *ALS5* insert of approximately 4.0 kb in size (3984 bp) from pGEX-6P-2 vector (4.9 kb). **(B)** Cloning of *ALS5-SS* in pGEX-6P-2 vector. *ALS5-SS* sequence was amplified by PCR (Table S1) and the amplicon was digested with BamHI and XhoI restriction enzymes. The restriction enzyme digested amplicon was ligated into similarly digested pGEX-6-P2 vector and the construct after ligation was confirmed by restriction enzyme digestion. Lane 1: Uncut plasmid after ligation of *ALS5-SS* in pGEX-6P-2 vector, Lane M: DNA molecular size marker, Lane 2: pGEX6P-2 vector with *ALS5-SS* sequence cloned and restricted with BamHI and XhoI enzymes, resulting in release of *ALS5-SS* insert of approximately 4.0 kb in size (4044 bp) from pGEX-6P-2 vector (4.9 kb).
(TIF)

Figure S2 **MALDI-TOF analysis of GST-Als5-SS.** Purified GST-Als5-SS was run on a 6% SDS polyacrylamide gel and stained using Coomassie Brilliant Blue R250. The band corresponding to the purified GST-Als5-SS protein was cut from the gel, trypsinized and taken for MALDI-TOF analysis. The matrix used was α-cyano-4-hydroxycinnamic acid. The data was analyzed using MASCOT software. The matched sequences within the Als5 sequence are shown in red and the gray bars indicate the various peptides that aligned with the sequence.
(TIF)

Figure S3 **ESI-MS analysis for GST-Als5-SS.** Purified GST-Als5-SS was run on a 6% SDS polyacrylamide gel and stained using Coomassie Brilliant Blue R250. The band corresponding to the purified GST-Als5-SS protein was cut from the gel, trypsinized and taken for ESI-MS analysis. The matched peptides within the Als5 sequence are shown in red in the lower panel.
(TIF)

Figure S4 **Intact mass of GST-Als5-SS using MALDI-TOF.** The purified GST-Als5-SS protein was analyzed using MALDI-TOF to determine the intact mass of the protein. A peak of 168.6 kDa, corresponding to the mass of GST-Als5-SS, was detected in the sample eluted from the glutathione-agarose column.
(TIF)

Figure S5 **Treatment of collagen type IV with N-Glycosidase F results in decrease in adherence of GST-Als5.** A flow diagram to describe the different steps and the various controls used in the assay. Collagen type IV (100 µl; 1 mg/ml) was immobilised overnight at 4°C in 96-well ELISA plate. The wells were then rinsed 5 times with PBS and incubated with N-glycosidase F (Roche) for 2 hours at 37^o^C in 50 mM sodium phosphate buffer (pH 8.0) containing 25 mM EDTA and 1% v/v β-mercaptoethanol. After washing 5 times with PBS, the subsequent steps were carried out as described in Experimental Procedures. GST-Als5 was incubated before the adherence was detected by using primary anti-GST antibody and anti-rabbit HRP-conjugated secondary antibody and is represented in this figure by the Absorbance at 650nm observed upon oxidation of the specific substrate of HRP. No Glycosidase: Only the incubation buffer of the enzyme was added. With glycosidase: 0.5 U glycosidase was added. All other wells were incubated at 37^o^C in incubation buffer. GST control: GST was added in place of GST-Als5; Buffer control: No immobilized collagen; Without primary Ab: The step where primary Ab was to be added was replaced with incubation with the buffer only. Nearly 25% drop in adherence of GST-Als5 to collagen type IV was observed after treatment of immobilised collagen with N-glycosidase F.
(TIF)

Figure S6 **TANGO prediction of propensity for secondary structure formation and β-aggregation in the unfolded sequence of Als5.** The sequence of Als5 minus the 17 residue N-terminal signal sequence and the GPI anchor attachment signal sequence was used for the analysis. Residues 325–329 in the N-terminal domain of Als5 show a very high propensity for β-sheet aggregation. No propensity for α-aggregation was predicted by this program for the Als5 sequence.
(TIF)

Figure S7 **Homology modelling of Als5Nt-peptide complexes.** (A) Low energy Rosetta docked model of Als5Nt bound to peptide ligand (EHAHTPR) colored green. (B) A cluster of top 150 ensembles shown here were chosen on the basis of all atom Rosetta score with an intramodel C_α_ r.m.s.d of less than 1 Angstrom. (C) Low energy Rosetta docked model of Als5Nt bound to the C-terminal peptide (KFISVALFFFL) from SS. The Als5Nt is colored in red, and the C-terminal peptide of SS is colored blue.
(TIF)

Figure S8 **Binding of GST-Als5 with different peptides.** A, C, E and G show representative fluorescence spectra obtained upon binding of GST-Als5 with the peptide EHAHTPR at 15^o^C, 27^o^C, 37^o^C and 47^o^C, respectively. B, D, F and H represent the binding plots of GST-Als5 with different peptides at 15^o^C, 27^o^C, 37^o^C and 47^o^C, respectively. Each data point gives the average of three independent measurements done in duplicates and the error bars denote standard deviations. Spectra 1–7 represent binding of peptides with GST-Als5 in the following order of increasing concentrations of the peptides: 1: no peptide; 2: 0.037 µM; 3: 0.074 µM; 4: 0.443 µM; 5: 0.804 µM; 6: 1.154 µM; 7: 1.5 µM.
(TIF)

Figure S9 **Binding of GST-Als5-SS with different peptides.** A, C, E and G panels show representative fluorescence spectra obtained upon binding of GST-Als5-SS with the peptide EHAHTPR at 15^o^C, 27^o^C, 37^o^C and 47^o^C, respectively. B, D, F and H represent the binding plots of GST-Als5-SS with different peptides at 15^o^C, 27^o^C, 37^o^C and 47^o^C, respectively. Each data point give the average of three independent measurements done in duplicates and the error bars denote standard deviations. Spectra 1–7 represent binding of peptides with GST-Als5-SS in the following order of increasing concentrations of the peptides: 1: no peptide; 2: 0.037 µM; 3: 0.074 µM; 4: 0.443 µM; 5: 0.804 µM; 6: 1.154 µM; 7: 1.5 µM.
(TIF)

Table S1 **Primer sequences used for cloning of** ***ALS5*** **and** ***ALS*** **5-SS region into pGEX-6P-2 vector.** The primers *ALS5* FP and *ALS5* RP were used to amplify *ALS5* gene from the genomic DNA of *C.albicans* strain CAI4. The primers *ALS5-SS* FP and *ALS5-SS* RP were used to amplify *ALS5-SS* sequence from the genomic DNA of *C.albicans* strain CAI4. The BamHI and XhoI restriction enzyme sites in the primer sequences, used for cloning, are shown in italics.
(DOCX)

Table S2 **Secondary structure predictions for the Als5 and Als5-SS proteins using CONTIN software** (DICHROWEB: http://dichroweb.cryst.bbk.ac.uk). The secondary structure content was predicted after subtraction of the GST spectrum from that of the respective fusion proteins.
(DOCX)

## References

[1] Maeda Y, Kinoshita T (2011). Structural remodeling, trafficking and functions of glycosylphosphatidylinositol-anchored proteins. Prog. Lipid Res.. 50,.

[2] Eisenhaber B, Maurer-Stroh S, Novatchkova M, Schneider G, Eisenhaber F (2003). Enzymes and auxiliary factors for GPI lipid anchor biosynthesis and post-translational transfer to proteins.. Bioessays 25,.

[3] Dalley JA, Bulleid NJ (2003). How does the translocon differentiate between hydrophobic sequences that form part of either a GPI (glycosylphosphatidylinositol)-anchor signal or a stop transfer sequence? Biochem. Soc. Trans.. 31,.

[4] Hoyer LL (2001). The *ALS* gene family of *Candida albicans*. Trends Microbiol.. 9,.

[5] Richard ML, Plaine A (2006). Comprehensive Analysis of Glycosylphosphatidylinositol-Anchored Proteins in *Candida albicans*.. Eukaryotic Cell 6,.

[6] Martinez-Lopez R (2004). The GPI-anchored protein CaEcm33p is required for cell wall integrity, morphogenesis and virulence in *Candida albicans*.. Microbiology 150,.

[7] Richard M, Ibata-Ombetta S, Dromer F, Bordon-Pallier F, Jouault T (2002). Complete glycosylphosphatidylinositol anchors are required in *Candida albicans* for full morphogenesis, virulence and resistance to macrophages.. Molecular Microbiology 44,.

[8] Hoyer LL, Green CB, Oh S-H, Zhao X (2008). Discovering the secrets of the *Candida albicans* agglutinin-like sequence (*ALS*) gene family–a sticky pursuit. Med. Mycol.. 46,.

[9] Gaur NK, Klotz SA, Henderson RL (1999). Overexpression of the *Candida albicans ALA1* gene in *Saccharomyces cerevisiae* results in aggregation following attachment of yeast cells to extracellular matrix proteins, adherence properties similar to those of *Candida albicans*. Infect. Immun.. 67,.

[10] Sheppard DC, Yeaman MR, Welch WH, Phan QT, Fu Y (2004). Functional and structural diversity in the Als protein family of *Candida albicans*. J. Biol. Chem.. 279,.

[11] Rauceo JM, De Armond R, Otoo H, Kahn PC, Klotz SA (2006). Threonine-rich repeats increase fibronectin binding in the *Candida albicans* adhesin Als5p.. Eukaryotic Cell 5,.

[12] Otoo HN, Lee KG, Qiu W, Lipke PN (2008). *Candida albicans* Als adhesins have conserved amyloid-forming sequences.. Eukaryotic Cell 7,.

[13] Ramsook CB, Tan C, Garcia MC, Fung R, Soybelman G (2010). Yeast cell adhesion molecules have functional amyloid-forming sequences.. Eukaryotic Cell 9,.

[14] Provencher SW, Glöckner J (1981). Estimation of globular protein secondary structure from circular dichroism.. Biochemistry 20,.

[15] Das R, Baker D (2008). Macromolecular modeling with rosetta. Annu. Rev. Biochem.. 77,.

[16] London N, Raveh B, Movshovitz-Attias D, Schueler-Furman O (2010). Can self-inhibitory peptides be derived from the interfaces of globular protein-protein interactions?. Proteins 78,.

[17] Salgado PS, Yan R, Taylor JD, Burchell L, Jones R (2011). Structural basis for the broad specificity to host-cell ligands by the pathogenic fungus *Candida albicans*. Proc. Natl. Acad. Sci.. U.S.A. 108,.

[18] Case DA, Cheatham TE, Darden T, Gohlke H, Luo R (2005). The Amber biomolecular simulation programs.. J Comput Chem 26,.

[19] Feng Q, Fang Z, Yan Z, Xing R, Xie L (2009). The structure-function relationship of MSI7, a matrix protein from pearl oyster *Pinctada fucata*. Acta Biochim. Biophys. Sin.. (Shanghai) 41,.

[20] Alonso R, Llopis I, Flores C, Murgui A, Timoneda J (2001). Different adhesins for type IV collagen on *Candida albicans*: identification of a lectin-like adhesin recognizing the 7S(IV) domain.. Microbiology (Reading, Engl.) 147,.

[21] Donohue DS, Ielasi FS, Goossens KVY, Willaert RG (2011). The N-terminal part of Als1 protein from *Candida albicans* specifically binds fucose-containing glycans. Mol. Microbiol.. 80,.

[22] Uversky VN (2002). Natively unfolded proteins: a point where biology waits for physics. Protein Sci.. 11,.

[23] Xue B, Dunbrack RL, Williams RW, Dunker AK, Uversky VN (2010). PONDR-FIT: a meta-predictor of intrinsically disordered amino acids. Biochim. Biophys.. Acta 1804,.

[24] Yan R, Simpson PJ, Matthews SJ, Cota E (2010). Backbone 1H, 15N, 13C and Ile, Leu, Val methyl chemical shift assignments for the 33.5 kDa N-terminal domain of *Candida albicans ALS1*.. Biomol NMR Assign 4,.

[25] Frank AT, Ramsook CB, Otoo HN, Tan C, Soybelman G (2010). Structure and function of glycosylated tandem repeats from *Candida albicans* Als adhesins.. Eukaryotic Cell 9,.

[26] Rousseau F, Schymkowitz J, Serrano L (2006). Protein aggregation and amyloidosis: confusion of the kinds?. Current Opinion in Structural Biology 16,.

[27] Fernandez-Escamilla A-M, Rousseau F, Schymkowitz J, Serrano L (2004). Prediction of sequence-dependent and mutational effects on the aggregation of peptides and proteins. Nat. Biotechnol.. 22,.

[28] Linding R, Schymkowitz J, Rousseau F, Diella F, Serrano L (2004). A comparative study of the relationship between protein structure and beta-aggregation in globular and intrinsically disordered proteins. J. Mol. Biol.. 342,.

[29] Klotz SA, Gaur NK, Lake DF, Chan V, Rauceo J (2004). Degenerate peptide recognition by *Candida albicans* adhesins Als5p and Als1p. Infect. Immun.. 72,.

[30] Zhao X, Pujol C, Soll DR, Hoyer LL (2003). Allelic variation in the contiguous loci encoding *Candida albicans ALS5*, *ALS1* and *ALS9*.. Microbiology (Reading, Engl.) 149,.

[31] Rauceo JM, Gaur NK, Lee K-G, Edwards JE, Klotz SA (2004). Global cell surface conformational shift mediated by a *Candida albicans* adhesin. Infect. Immun.. 72,.

[32] Klotz SA, Gaur NK, De Armond R, Sheppard D, Khardori N (2007). *Candida albicans* Als proteins mediate aggregation with bacteria and yeasts. Med. Mycol.. 45,.

[33] Almeida A, Layton M, Karadimitris A (2009). Inherited glycosylphosphatidyl inositol deficiency: a treatable CDG. Biochim. Biophys.. Acta 1792,.

[34] Jost CR, Gaillard ML, Fransen JA, Daha MR, Ginsel LA (1991). Intracellular localization of glycosyl-phosphatidylinositol-anchored CD67 and FcRIII (CD16) in affected neutrophil granulocytes of patients with paroxysmal nocturnal hemoglobinuria.. Blood 78,.

[35] Singh N, Singleton D, Tartakoff AM (1991). Anchoring and degradation of glycolipid-anchored membrane proteins by L929 versus by LM-TK- mouse fibroblasts: implications for anchor biosynthesis. Mol. Cell. Biol.. 11,.

