# Supplementary figures and images for: The GPI Anchor Signal Sequence Dictates the Folding and Functionality of the Als5 Adhesin from Candida albicans
Source: PLoS One. 2012 Apr 11;7(4):e35305. doi: 10.1371/journal.pone.0035305 (PMC3324464; doi:10.1371/journal.pone.0035305)

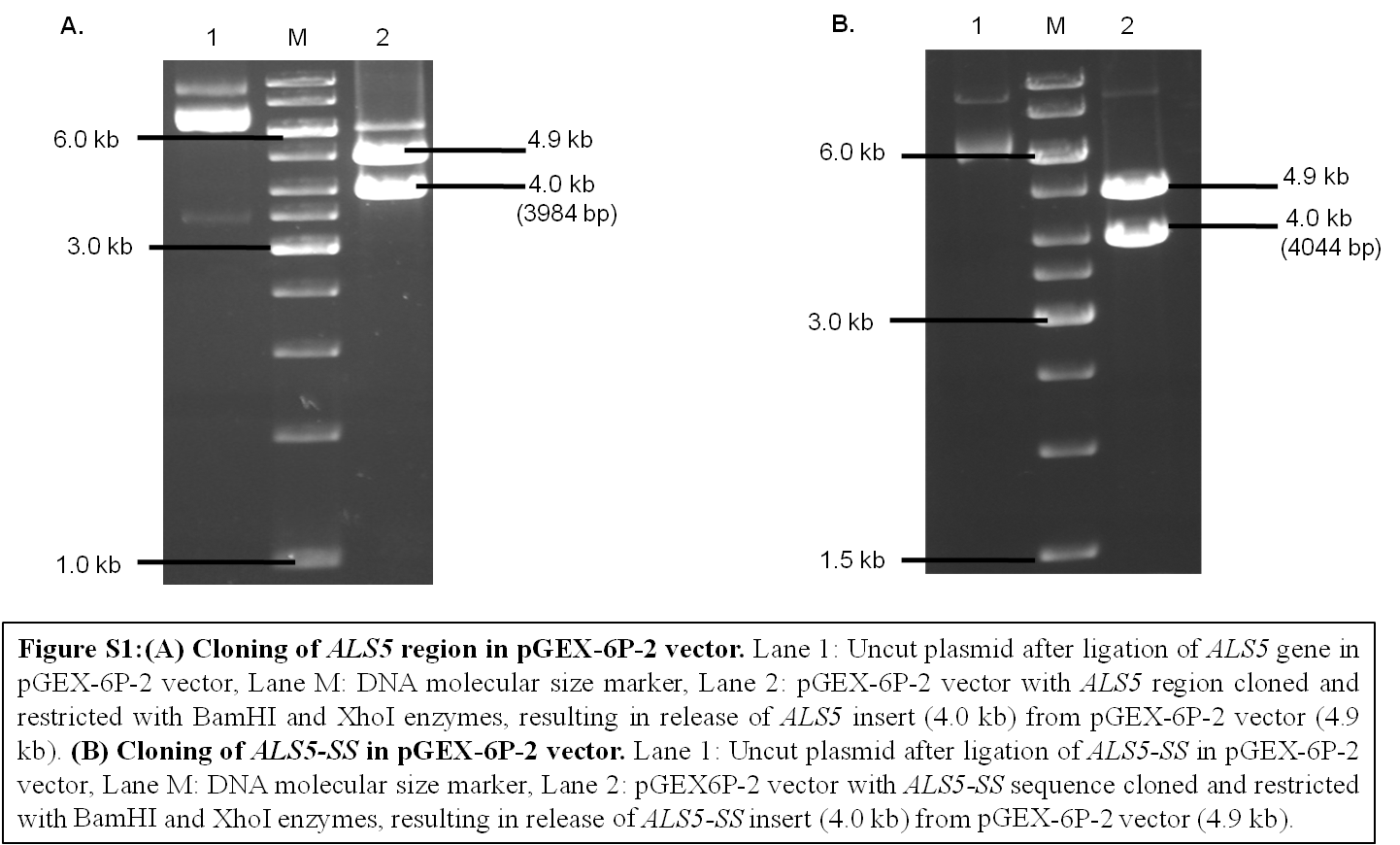

Supplement: Figure S1 — Cloning of ALS5 and ALS5-SS regions in pGEX-6-P2 vector. (A) Cloning of ALS5 region in pGEX-6P-2 vector. ALS5 sequence was amplified by PCR (Table S1) and the amplicon was digested with BamHI and XhoI restriction enzymes. The restriction enzyme digested amplicon was ligated into similarly digested pGEX-6-P2 vector and the construct after ligation was confirmed by restriction enzyme digestion. Lane 1: Uncut plasmid after ligation of ALS5 gene in pGEX-6P-2 vector, Lane M: DNA molecular size marker, Lane 2: pGEX-6P-2 vector with ALS5 region cloned and restricted with BamHI and XhoI enzymes, resulting in release of ALS5 insert of approximately 4.0 kb in size (3984 bp) from pGEX-6P-2 vector (4.9 kb). (B) Cloning of ALS5-SS in pGEX-6P-2 vector. ALS5-SS sequence was amplified by PCR (Table S1) and the amplicon was digested with BamHI and XhoI restriction enzymes. The restriction enzyme digested amplicon was ligated into similarly digested pGEX-6-P2 vector and the construct after ligation was confirmed by restriction enzyme digestion. Lane 1: Uncut plasmid after ligation of ALS5-SS in pGEX-6P-2 vector, Lane M: DNA molecular size marker, Lane 2: pGEX6P-2 vector with ALS5-SS sequence cloned and restricted with BamHI and XhoI enzymes, resulting in release of ALS5-SS insert of approximately 4.0 kb in size (4044 bp) from pGEX-6P-2 vector (4.9 kb). (TIF) [file pone.0035305.s001.tif]

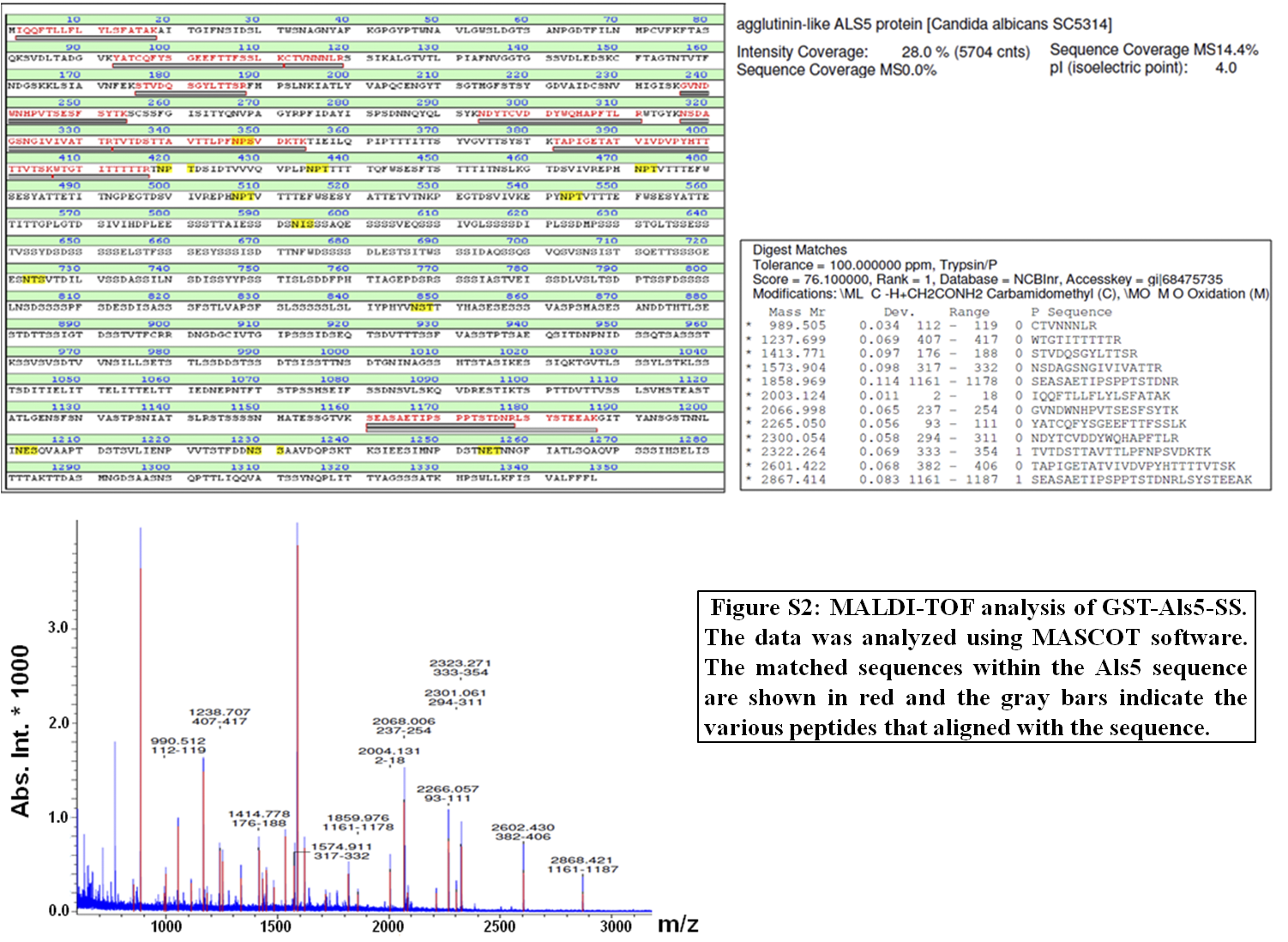

Supplement: Figure S2 — MALDI-TOF analysis of GST-Als5-SS. Purified GST-Als5-SS was run on a 6% SDS polyacrylamide gel and stained using Coomassie Brilliant Blue R250. The band corresponding to the purified GST-Als5-SS protein was cut from the gel, trypsinized and taken for MALDI-TOF analysis. The matrix used was α-cyano-4-hydroxycinnamic acid. The data was analyzed using MASCOT software. The matched sequences within the Als5 sequence are shown in red and the gray bars indicate the various peptides that aligned with the sequence. (TIF) [file pone.0035305.s002.tif]

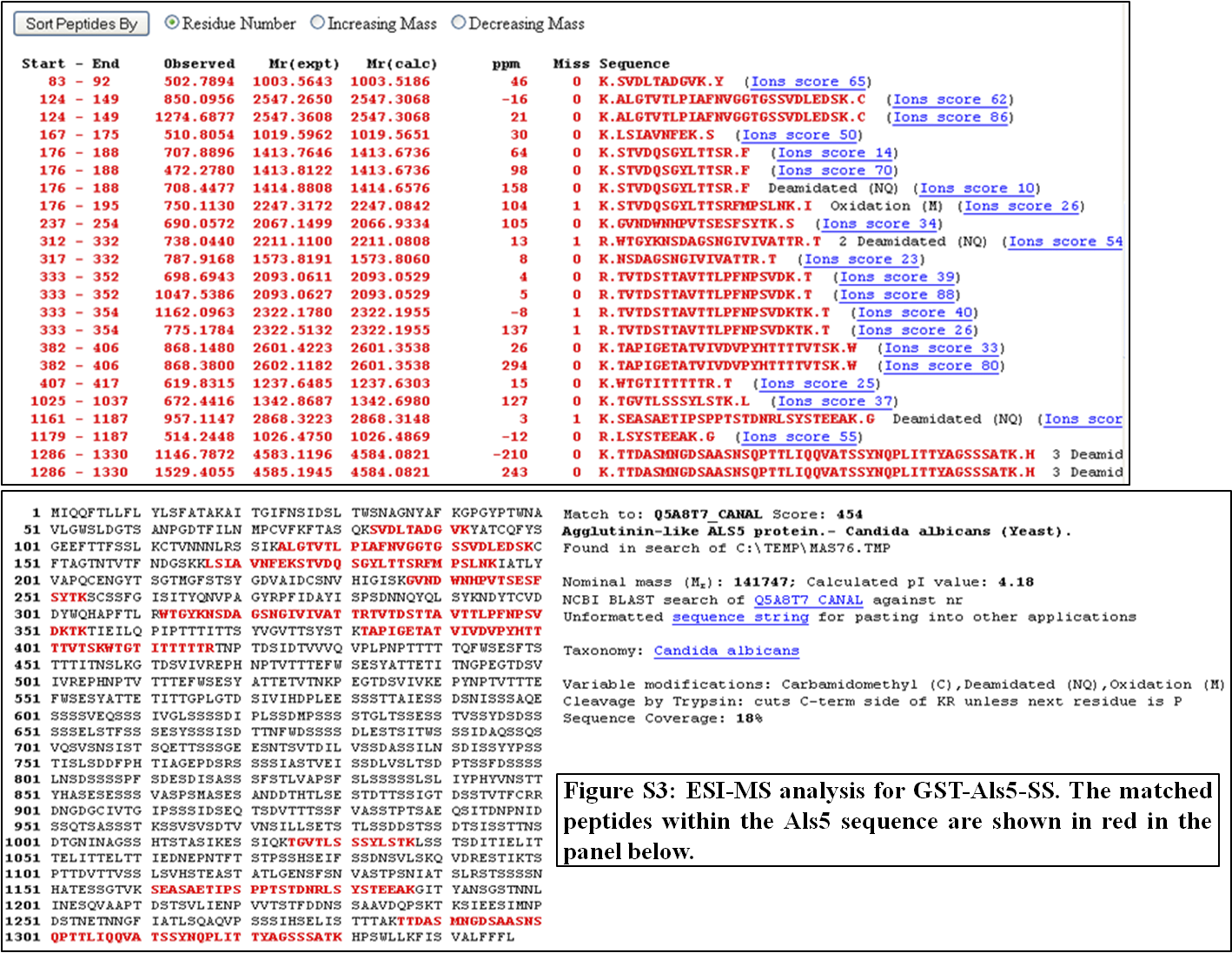

Supplement: Figure S3 — ESI-MS analysis for GST-Als5-SS. Purified GST-Als5-SS was run on a 6% SDS polyacrylamide gel and stained using Coomassie Brilliant Blue R250. The band corresponding to the purified GST-Als5-SS protein was cut from the gel, trypsinized and taken for ESI-MS analysis. The matched peptides within the Als5 sequence are shown in red in the lower panel. (TIF) [file pone.0035305.s003.tif]

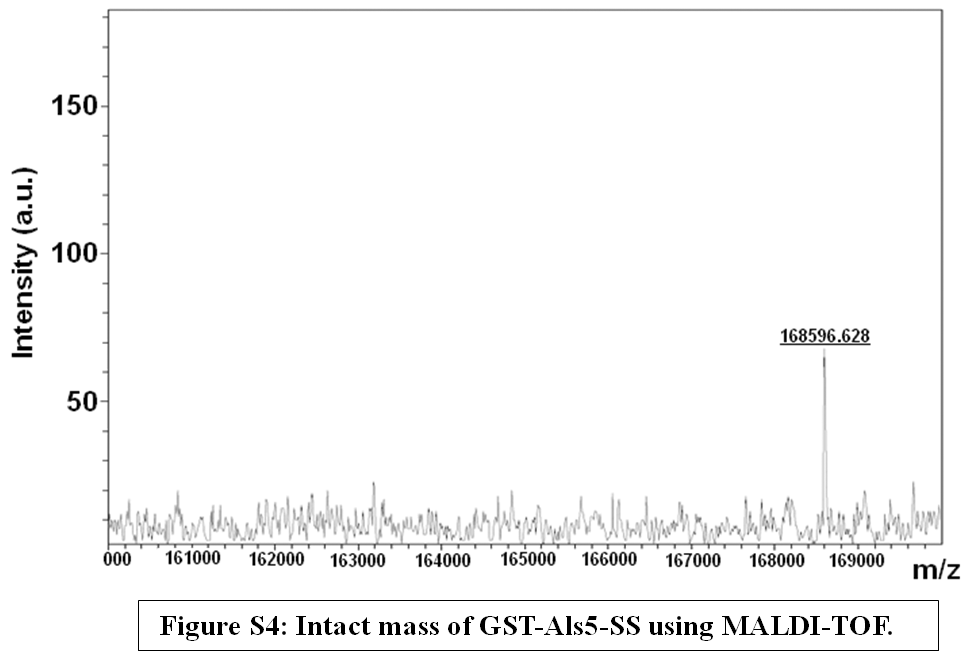

Supplement: Figure S4 — Intact mass of GST-Als5-SS using MALDI-TOF. The purified GST-Als5-SS protein was analyzed using MALDI-TOF to determine the intact mass of the protein. A peak of 168.6 kDa, corresponding to the mass of GST-Als5-SS, was detected in the sample eluted from the glutathione-agarose column. (TIF) [file pone.0035305.s004.tif]

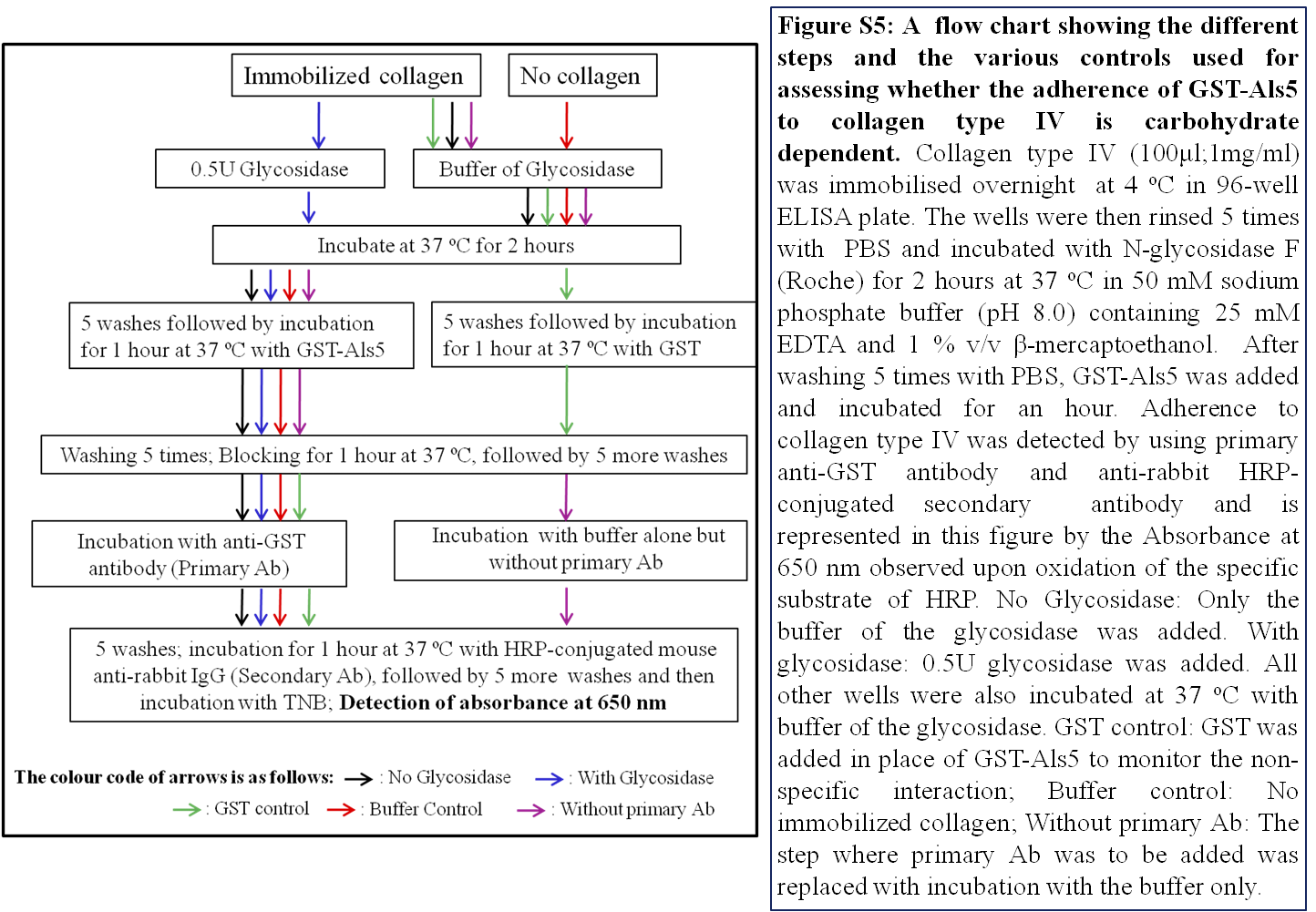

Supplement: Figure S5 — Treatment of collagen type IV with N-Glycosidase F results in decrease in adherence of GST-Als5. A flow diagram to describe the different steps and the various controls used in the assay. Collagen type IV (100 µl; 1 mg/ml) was immobilised overnight at 4°C in 96-well ELISA plate. The wells were then rinsed 5 times with PBS and incubated with N-glycosidase F (Roche) for 2 hours at 37oC in 50 mM sodium phosphate buffer (pH 8.0) containing 25 mM EDTA and 1% v/v β-mercaptoethanol. After washing 5 times with PBS, the subsequent steps were carried out as described in Experimental Procedures. GST-Als5 was incubated before the adherence was detected by using primary anti-GST antibody and anti-rabbit HRP-conjugated secondary antibody and is represented in this figure by the Absorbance at 650nm observed upon oxidation of the specific substrate of HRP. No Glycosidase: Only the incubation buffer of the enzyme was added. With glycosidase: 0.5 U glycosidase was added. All other wells were incubated at 37oC in incubation buffer. GST control: GST was added in place of GST-Als5; Buffer control: No immobilized collagen; Without primary Ab: The step where primary Ab was to be added was replaced with incubation with the buffer only. Nearly 25% drop in adherence of GST-Als5 to collagen type IV was observed after treatment of immobilised collagen with N-glycosidase F. (TIF) [file pone.0035305.s005.tif]

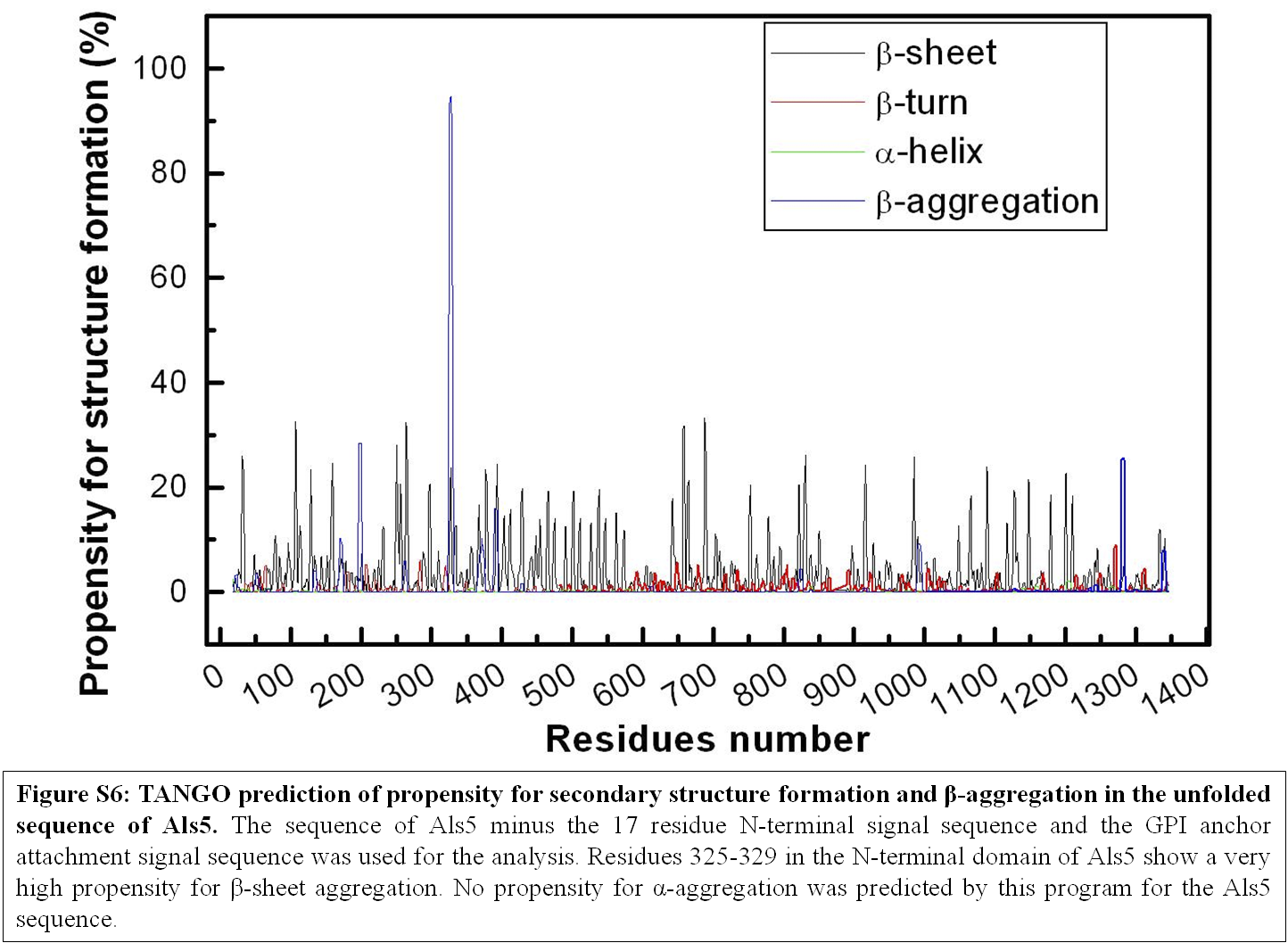

Supplement: Figure S6 — TANGO prediction of propensity for secondary structure formation and β-aggregation in the unfolded sequence of Als5. The sequence of Als5 minus the 17 residue N-terminal signal sequence and the GPI anchor attachment signal sequence was used for the analysis. Residues 325–329 in the N-terminal domain of Als5 show a very high propensity for β-sheet aggregation. No propensity for α-aggregation was predicted by this program for the Als5 sequence. (TIF) [file pone.0035305.s006.tif]

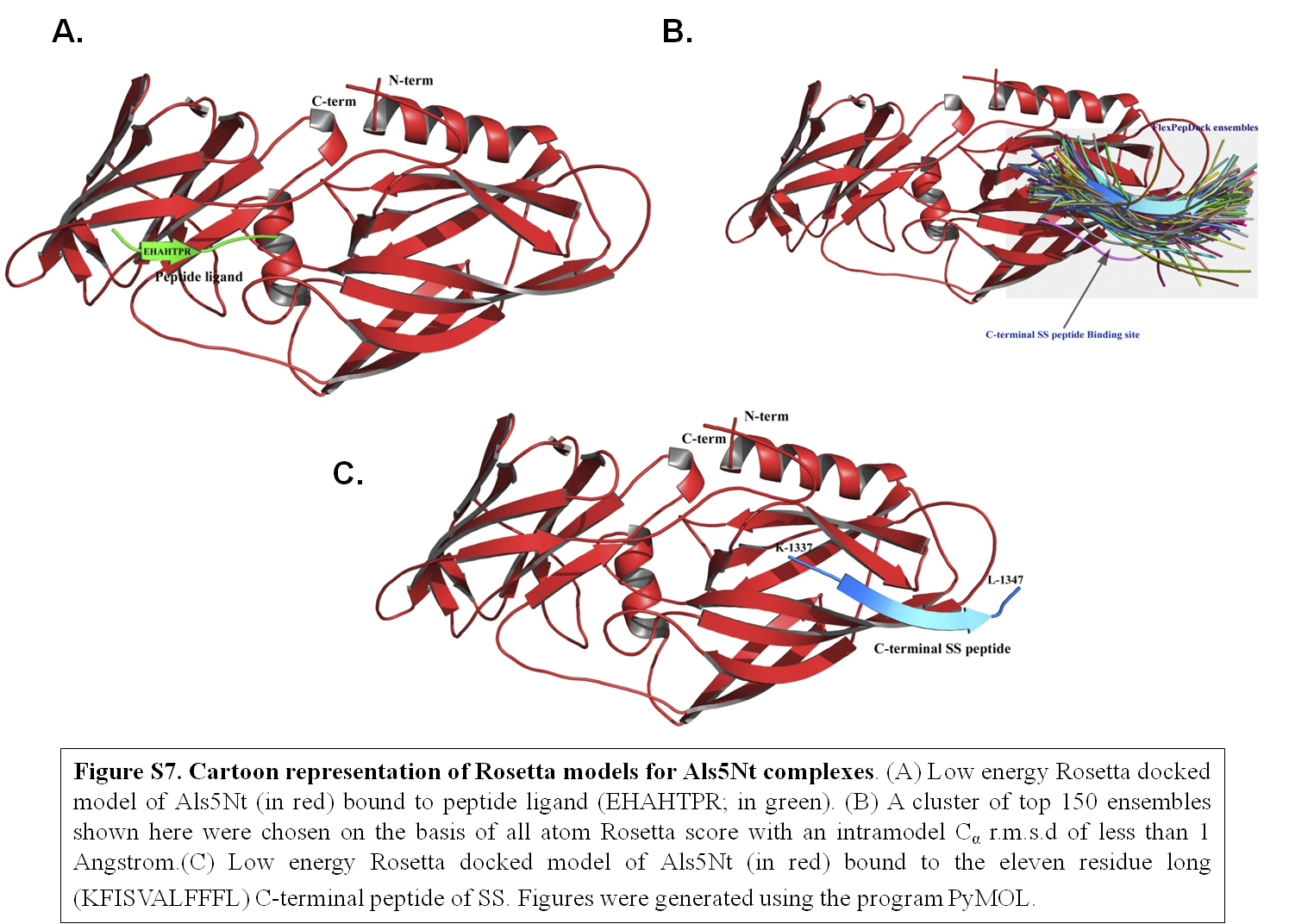

Supplement: Figure S7 — Homology modelling of Als5Nt-peptide complexes. (A) Low energy Rosetta docked model of Als5Nt bound to peptide ligand (EHAHTPR) colored green. (B) A cluster of top 150 ensembles shown here were chosen on the basis of all atom Rosetta score with an intramodel Cα r.m.s.d of less than 1 Angstrom. (C) Low energy Rosetta docked model of Als5Nt bound to the C-terminal peptide (KFISVALFFFL) from SS. The Als5Nt is colored in red, and the C-terminal peptide of SS is colored blue. (TIF) [file pone.0035305.s007.tif]

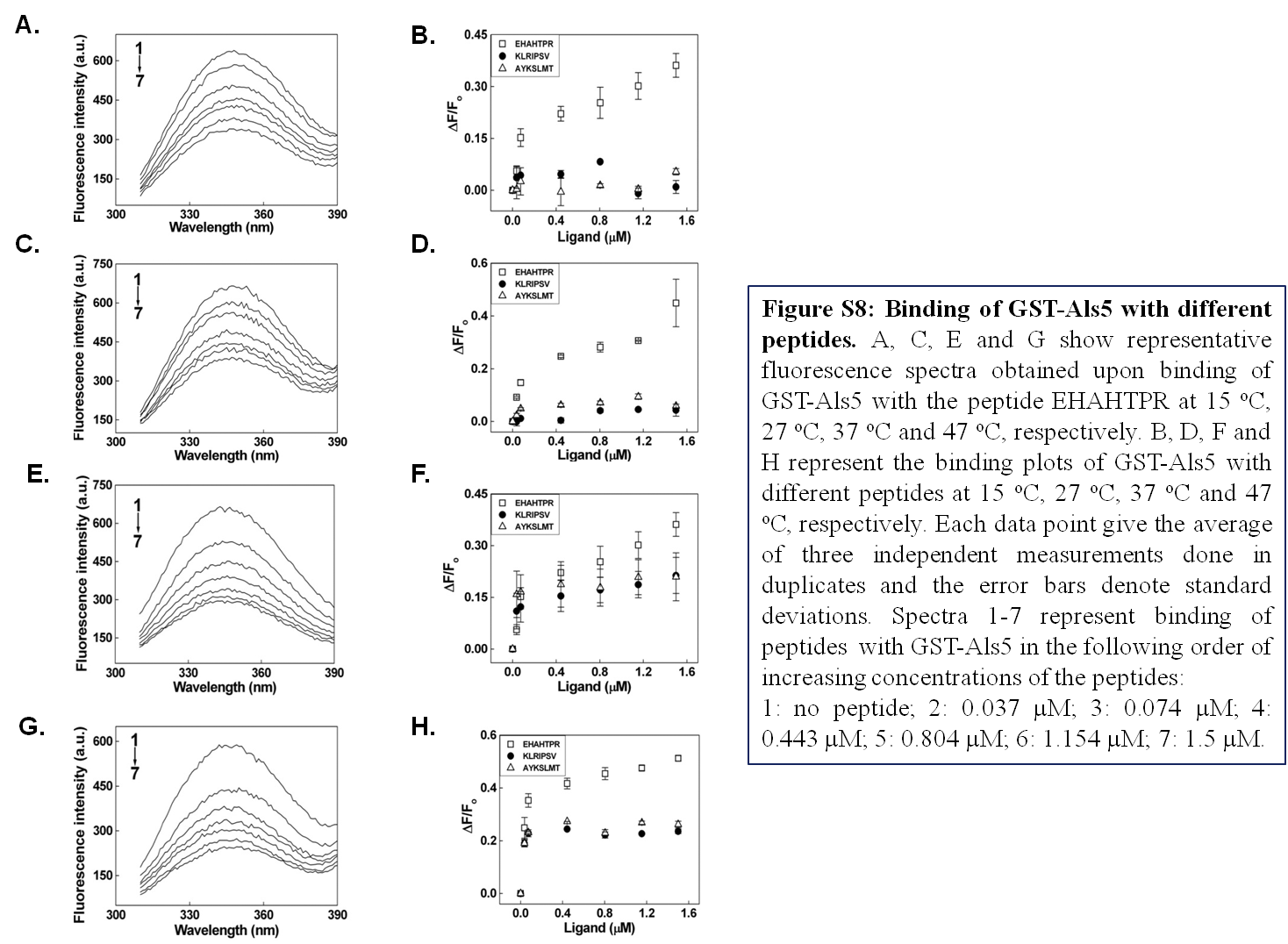

Supplement: Figure S8 — Binding of GST-Als5 with different peptides. A, C, E and G show representative fluorescence spectra obtained upon binding of GST-Als5 with the peptide EHAHTPR at 15oC, 27oC, 37oC and 47oC, respectively. B, D, F and H represent the binding plots of GST-Als5 with different peptides at 15oC, 27oC, 37oC and 47oC, respectively. Each data point gives the average of three independent measurements done in duplicates and the error bars denote standard deviations. Spectra 1–7 represent binding of peptides with GST-Als5 in the following order of increasing concentrations of the peptides: 1: no peptide; 2: 0.037 µM; 3: 0.074 µM; 4: 0.443 µM; 5: 0.804 µM; 6: 1.154 µM; 7: 1.5 µM. (TIF) [file pone.0035305.s008.tif]

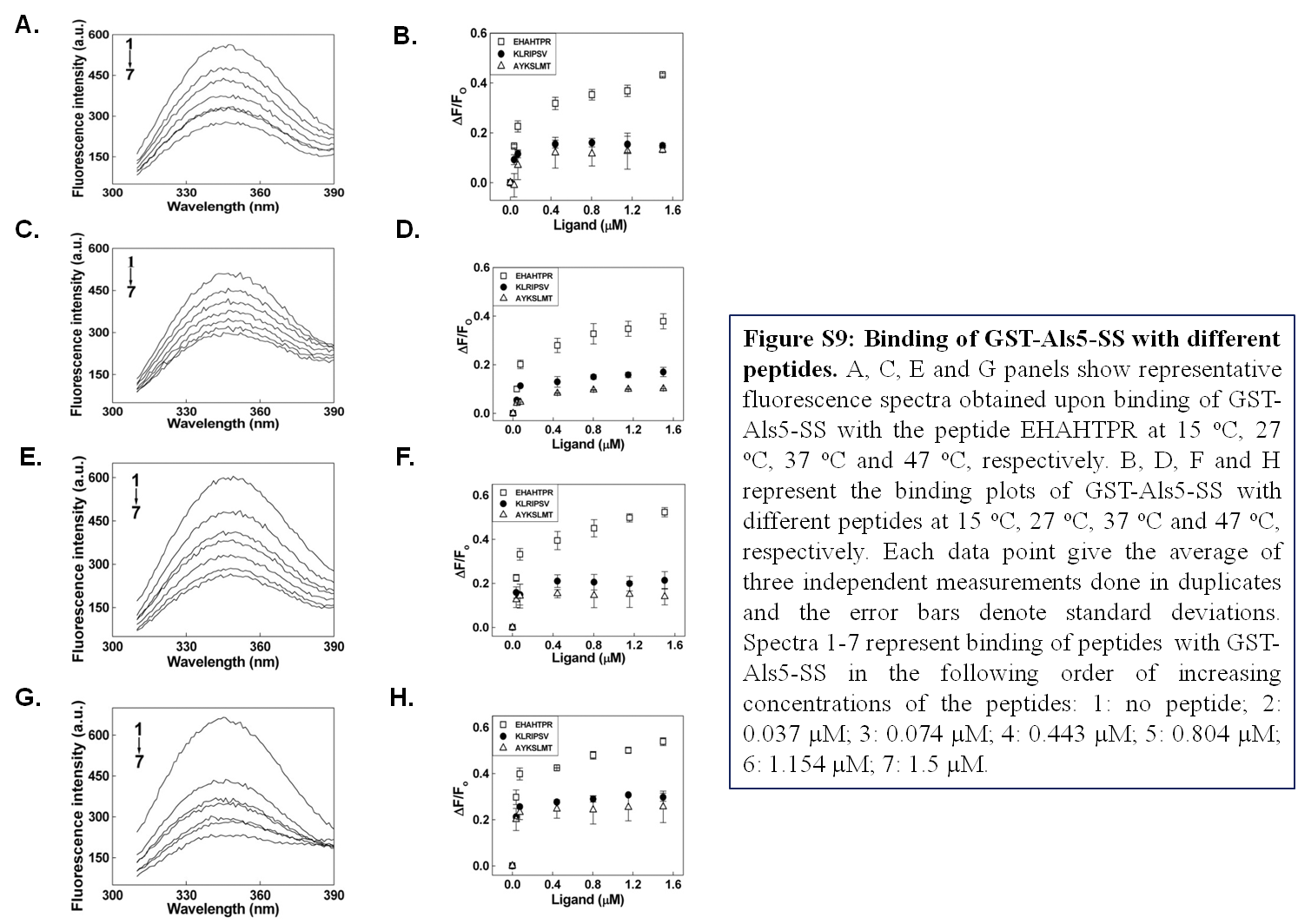

Supplement: Figure S9 — Binding of GST-Als5-SS with different peptides. A, C, E and G panels show representative fluorescence spectra obtained upon binding of GST-Als5-SS with the peptide EHAHTPR at 15oC, 27oC, 37oC and 47oC, respectively. B, D, F and H represent the binding plots of GST-Als5-SS with different peptides at 15oC, 27oC, 37oC and 47oC, respectively. Each data point give the average of three independent measurements done in duplicates and the error bars denote standard deviations. Spectra 1–7 represent binding of peptides with GST-Als5-SS in the following order of increasing concentrations of the peptides: 1: no peptide; 2: 0.037 µM; 3: 0.074 µM; 4: 0.443 µM; 5: 0.804 µM; 6: 1.154 µM; 7: 1.5 µM. (TIF) [file pone.0035305.s009.tif]
