# Supplementary material for: The GPI Anchor Signal Sequence Dictates the Folding and Functionality of the Als5 Adhesin from Candida albicans
Source: PLoS One. 2012 Apr 11;7(4):e35305. doi: 10.1371/journal.pone.0035305 (PMC3324464; doi:10.1371/journal.pone.0035305)
Supplement: Table S1 — Primer sequences used for cloning of ALS5 and ALS 5-SS region into pGEX-6P-2 vector. The primers ALS5 FP and ALS5 RP were used to amplify ALS5 gene from the genomic DNA of C.albicans strain CAI4. The primers ALS5-SS FP and ALS5-SS RP were used to amplify ALS5-SS sequence from the genomic DNA of C.albicans strain CAI4. The BamHI and XhoI restriction enzyme sites in the primer sequences, used for cloning, are shown in italics. (DOCX) [file pone.0035305.s010.docx]

**Table S1: Primer sequences used for cloning of *ALS5* and *ALS*5-SS region into pGEX-6P-2 vector.** The primers *ALS5* FP and *ALS5* RP were used to amplify *ALS5* gene from the genomic DNA of *C.albicans* strain CAI4. The primers *ALS5-SS* FP and *ALS5-SS* RP were used to amplify *ALS5-SS* sequence from the genomic DNA of *C.albicans* strain CAI4. The BamHI and XhoI restriction enzyme sites, used for cloning, are shown in italics.

| Primer name | Primer Sequence |
| --- | --- |
| *ALS5* FP | **5' GCG*GGATCC*ATGATTCAACAATTTACATTGTTATTC 3'** |
| *ALS5* RP | **5' CGC*CTCGAG*TCAGGATGAAGATCCGGCATAAG 3'** |
| *ALS5-SS* FP | **5' GCG*GGATCC*ATGATTCAACAATTTACATTGTTATTC 3'** |
| *ALS5-SS* RP | **5' GCG*CTCGAG*TCATAGAAAGAAGAATAATGCAACG 3'** |
