# Supplementary material for: The GPI Anchor Signal Sequence Dictates the Folding and Functionality of the Als5 Adhesin from Candida albicans
Source: PLoS One. 2012 Apr 11;7(4):e35305. doi: 10.1371/journal.pone.0035305 (PMC3324464; doi:10.1371/journal.pone.0035305)
Supplement: Table S2 — Secondary structure predictions for the Als5 and Als5-SS proteins using CONTIN software (DICHROWEB: http://dichroweb.cryst.bbk.ac.uk). The secondary structure content was predicted after subtraction of the GST spectrum from that of the respective fusion proteins. (DOCX) [file pone.0035305.s011.docx]

**Table S2:** Secondary structure predictions for the Als5 and Als5-SS proteins using CONTIN software (DICHROWEB: http://dichroweb.cryst.bbk.ac.uk). The secondary structure content was predicted after subtraction of the GST spectrum from that of the respective fusion proteins.

| Result | Helix1 | Helix2 | Strand1 | Strand2 | Turns | Unordered | Total |
| --- | --- | --- | --- | --- | --- | --- | --- |
| Als5 | | | | | | | |
| Closest matching solution with all proteins | 0.000 | 0.092 | 0.000 | 0.254 | 0.438 | 0.216 | 1 |
| Average of all matching solutions | 0.000 | 0.064 | 0.198 | 0.185 | 0.426 | 0.126 | 0.999 |
| Als5-SS | | | | | | | |
| Closest matching solution with all proteins | 0.470 | 0.266 | 0.000 | 0.000 | 0.000 | 0.264 | 1 |
| Average of all matching solutions | 0.517 | 0.085 | 0.118 | 0.045 | 0.000 | 0.235 | 1 |
